# Neural circuits encode prior knowledge of temporal statistics

**DOI:** 10.1038/s41593-026-02255-7

**Published:** 2026-04-07

**Authors:** Julius Koppen, Ilse Klinkhamer, Marit Runge, Lucas Bayones, Devika Narain

**Affiliations:** 1 Donders Center for Neuroscience, Donders Institute, Radboud University, Nijmegen, The Netherlands; 2 Department of Neuroscience, Erasmus University Medical Center, Rotterdam, The Netherlands; 3 Instituto de Fisiología Celular, Departamento de Neurociencia Cognitiva, Universidad Nacional Autónoma de México, Mexico City, Mexico

**Keywords:** Classical conditioning, Neural circuits, Cognitive neuroscience

## Abstract

The brain must infer the state of the external world despite the inherent uncertainty of its sensory inputs and internal processes. Under conditions of heightened uncertainty, it increasingly relies on prior knowledge, derived from accumulated experience with the regularities and statistical structures of the environment. This principle has been formalized by Bayesian inference theories, which are supported by substantial evidence from both behavioral and neuroscience studies. However, direct evidence for the existence of prior knowledge in the brain, and for the encoding of environmental statistics by neural circuits, remains limited. Here we show that cerebellar circuits learn the prior probability distribution of temporal variables during eyeblink conditioning in mice and encode these representations in Purkinje cell simple and complex spike signaling. We further demonstrate that Purkinje cells are involved in eliciting predictive motor behaviors, such as the conditioned eyeblink response, that also reflect the statistics of the experimentally imposed prior distribution of the stimulus. Computational modeling of these results indicates the juxtaposition of counteracting long-term plasticity mechanisms by which cerebellar Purkinje cells could acquire prior knowledge that is shaped by the statistics of different probability distributions. Our results suggest that the cerebellar circuitry may be uniquely poised to learn the probability of events in the world and internalize these as prior knowledge. These findings advance understanding of how neural computations could implement Bayesian inference.

## Abstract (web summary)

This study shows that cerebellar circuits learn and encode prior probabilities of event timing. Cell-type-specific neural activity reflects environmental statistics and guides predictive motor behavior, providing support for neural Bayesian inference.

## Main

Information received by the brain about the environment is frequently ambiguous, degraded by noise and subject to transformation as it propagates through neural circuits, resulting in inherently uncertain internal representations. Given the pervasive nature of such uncertainty in neural processing, the brain would need to rely on its prior experience with the regularity and probability of variables in the world to guide the predictive behaviors it generates. This foundational principle has been formalized within Bayesian inference frameworks that are increasingly influential across both behavioral and neuroscientific research^1–6^. Having a form of memory that encodes the prior probability of environmental variables could be beneficial to survival in many instances, ranging from the most probable angle of approach of a local predator to the most likely places that we encounter traffic on daily commutes. Little, however, is known about neural circuit mechanisms responsible for learning the statistics of probabilistic variables in the world to guide anticipatory behavior.

Prior knowledge plays a crucial role in Bayesian inference; however, it remains unclear how and where this knowledge is represented in the brain, even though previous studies showed both Bayes-consistent behavior and neural correlates^7–17^. For instance, we know of cortical representations underlying Bayes-like behaviors in sensory^6,18^, cognitive^15^ and motor systems^14^. The question then arises, if humans^9,19^, non-human primates^15^ and rodents^20^ can learn a variety of tasks with probabilistic stimuli, what are the neural circuit mechanisms by which such probability distributions are acquired and used to generate predictions in an uncertain world?

Among brain structures, the cerebellum appears particularly well suited to learning prior distributions of temporal statistics. The cerebellar cortical machinery has long been implicated in pure timing behaviors^21,22^ alongside various temporally dependent sensorimotor behaviors^23–34^. Additionally, numerous theoretical frameworks have proposed that the cerebellum serves as a substrate for internal models^35–37^, motor processes^37^, ocular function^38^, vestibular regulation^39,40^ and inertial perception^41^. Notably, the principal output neuron of the cerebellar cortex, the Purkinje cell, exhibits two distinct firing modes: low-frequency complex spikes (CSpks) and high-frequency simple spikes (SSpks). This dual firing capability may allow Purkinje cells to integrate multimodal inputs and facilitate the encoding of diverse temporal contingencies^42^.

Here we show that cerebellar Purkinje cells can learn the temporal statistics of time intervals drawn from distinct probability distributions. Specifically, we demonstrate that cerebellar cortical populations support the emergence of adaptive, predictive eyeblink behaviors that track dynamic changes in the probabilistic structure of temporal stimuli. We also propose a computational mechanism that hypothesizes a role for the juxtaposition of long-term depression (LTD) and long-term potentiation (LTP) mechanisms to enable the learning of probabilistic temporal statistics by Purkinje cells. Together, these results suggest that the cerebellum is ideally poised to learn the probabilistic statistics of behaviorally relevant events.

## Results

### Predictive eyelid behavior modulates with changes in temporal statistics

**Fig. 1:**
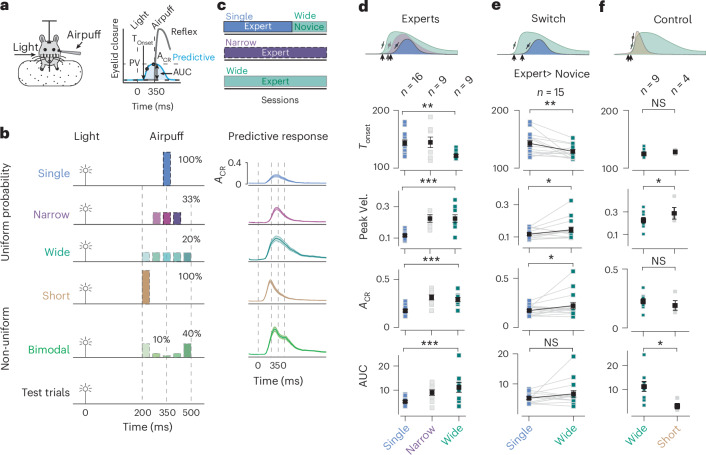
Prior probability distributions shape predictive eyeblink traces. **a**, Left, head-fixed mice learn temporal delays between a light flash and a periocular airpuff. Right, the airpuff elicits reflexive eye closure (gray) on paired trials, but, after training, the eye closes preemptively (blue), even in the absence of the airpuff (on test trials), known as a CR (or a predictive response), which is evaluated using *T*_onset_, *A*_CR_, AUC and peak velocity (arrows). **b**, Stimuli are sampled from different probability distributions: Single represents a probability of 1 (100%) of the airpuff on paired trials at 350 ms (blue); Narrow represents a uniform probability of 0.33 (33% chance, purple); and Wide represents a uniform probability of 0.2 on paired trials (20% chance, cyan). The Short condition has a probability of 1 for the 200-ms interval (brown). In the Bimodal condition, the stimulus intervals are non-uniform (40% chance for 200 ms and 500 ms, 10% chance for 275 ms and 425 ms, green). Right, examples of the average conditioned eyeblink response amplitude (*A*_CR_) in a subject on test trials (airpuff omitted). **c**, Expert groups were trained on prior conditions Single (*n* = 16 mice), Narrow (*n* = 9), Wide (*n* = 9), Short (*n* = 4) and Bimodal (*n* = 5). In the switch group, mice were switched from Single to Wide (*n* = 15). **d**, Metric comparisons of one-way ANOVA statistics for *T*_onset_ (*F*_2,33_ = 6.13, *P* = 0.005), peak velocity (*F*_2,33_ = 18.6, *P* = 4 × 10^−6^), *A*_CR_ *F*_2,33_ = 12.14, *P* = 0.0001) and area under the eyeblink response curve (AUC; *F*_2,33_ = 8.95, *P* = 0.0008) across three expert groups: Single (blue), Narrow (purple) and Wide (cyan). Arrows and tangent lines respectively indicate *T*_onset_ and peak velocity for each prior. **e**, Comparisons with *t*-statistics within the switch group where each mouse switched from Single (blue) to Wide (cyan) for *T*_onset_ (#, *t*_14_ = −2.98, *P* = 0.005), peak velocity (#, *t*_14_ = 2.06, *P* = 0.03), *A*_CR_ (#, *t*_14_ = 1.81, *P* = 0.05) and AUC (#, *t*_14_ = 1.36, *P* = 0.1). **f**, A control comparison with *t*-statistics of the Wide experts (cyan) with the Short experts (brown) to evaluate if the full distribution was considered by evaluating metrics *T*_onset_ (##, *t*_11_ = 0.79, *P* = 0.4), peak velocity (#, *t*_11_ = 2.38, *P* = 0.02), *A*_CR_ (##, *t*_11_ = 0.82, *P* = 0.4) and AUC (#, *t*_11 _= 2.48, *P* = 0.02). In all cases, black squares represent averages; error bars indicate standard error. # indicates one-sided *t*-test; ## indicates two-sided *t*-test. *P < 0.05; **P < 0.01; ***P < 0.001. NS, not significant; Peak Vel., peak velocity.

To study how prior probability distributions influence predictive behavior, we developed a variant of the trace eyeblink conditioning paradigm. In the classical trace eyeblink conditioning paradigm, animals learn a fixed temporal association between a conditioned stimulus (typically a light) and an unconditioned stimulus (a periocular airpuff), which elicits a reflexive eyelid closure (Fig. 1a). After repeated pairings, the eyelid begins to exhibit a conditioned response (CR, also referred to as a predictive response), characterized by anticipatory eye closure occurring at the expected interval, even in the absence of the airpuff (Fig. 1a, right). We developed a probabilistic variant of trace conditioning where the interval between the light and the airpuff on each trial was sampled from one of several experimentally defined prior distributions: Single, Narrow, Wide, Short or Bimodal (Fig. 1b). Mice were either trained exclusively on one of these distributions throughout the experiment (‘expert’ mice) or initially trained on the Single prior and subsequently switched to the Wide prior (‘switch’ mice) (Fig. 1c).

**Extended Data Fig. 1:**
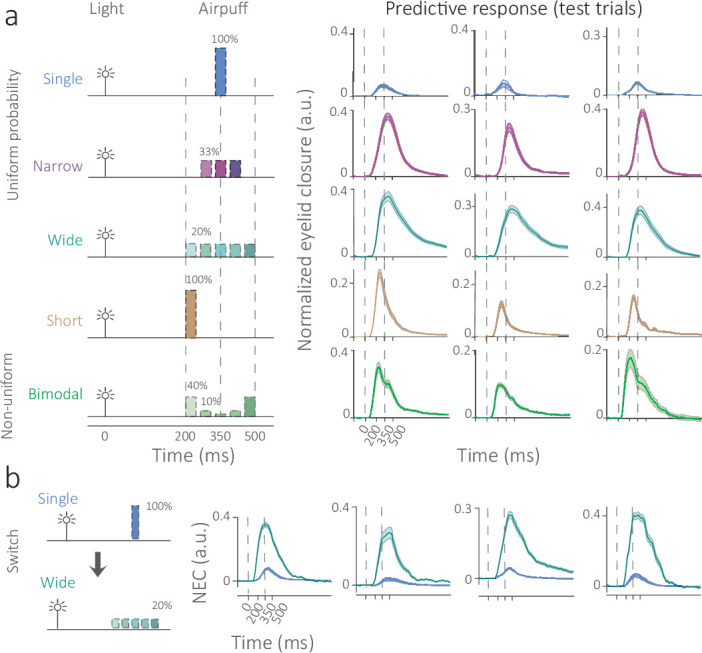
Examples of eyeblink traces for different prior probability distributions. **a**) Examples of average behavioral traces from individual subjects on test trials (airpuff is omitted) for the Single (blue), Narrow (purple), Wide (cyan), Short (brown), Bimodal (green) prior conditions. Solid lines represent averages, shaded areas represent standard error of the mean. **b**) Same subject switched from Single (blue) to Wide (cyan) prior condition. Average eyelid closure traces for four subjects. Solid lines represent averages, shaded areas represent standard error of the mean.

We hypothesized that the shape of the prior probability distribution of stimuli would influence the predictive eyeblink response and that this effect would be reflected both in the form of the eyeblink itself and in its kinematic properties. To test this, we analyzed blink-related metrics in ‘expert’ mice trained on distinct temporal distributions, using randomly interleaved test trials in which the unconditioned stimulus (airpuff) was omitted, thereby isolating purely anticipatory behavior (Fig. 1b and Extended Data Fig. 1a). The measured parameters included onset time (*T*_onset_), marking the earliest time of eyelid closure; peak velocity of the initial eyelid closure; amplitude of the CR (*A*_CR_); and the area under the curve (AUC) of the CR (Fig. 1a, right).

**Extended Data Fig. 2:**
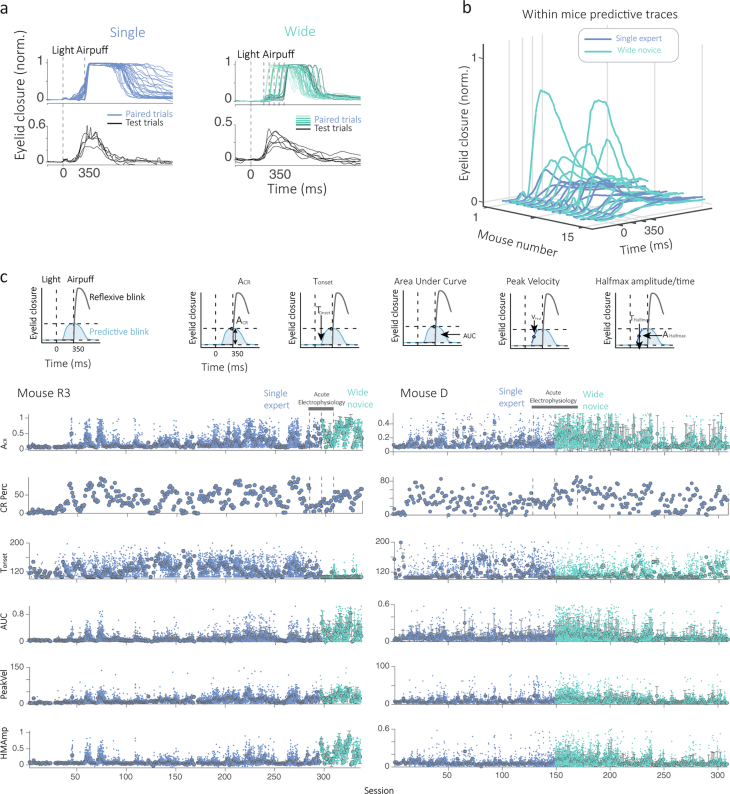
Example traces for switch mice and progression of eyeblink trace metrics over the course of training. **a**) Normalized trial-wise eyelid closure traces for Single expert paired (blue) and test trials (black) and Wide expert paired (cyan) and test trials (black). **b**) Test traces for eyelid closure of all mice (N = 15) that transitioned from the Single (blue) to Wide (cyan) condition. **c**) Above: Description of various metrics used to quantify the predictive eyeblink. Below: Session-wise progression of learning metrics for two switch mice that transitioned from Single to Wide. The period where electrophysiology was performed is also indicated.

We found that behavioral metrics varied systematically with the statistical properties of the underlying temporal distributions. For example, when the earliest possible interval in the distribution occurred sooner, *T*_onset_ correspondingly decreased, indicating a shift in anticipatory timing (Fig. 1d). Also, the *A*_CR_ increased under wider distributions compared to the Single condition (Fig. 1d). The peak velocity and AUC also increased (Fig. 1d) for wider distributions, potentially extending the compensatory mechanism of the conditioned eyeblink to the different probabilities of airpuff occurrences. The same adaptations were studied in the ‘switch’ mice as they transitioned from the Single to the Wide prior (*n* = 15 mice; Fig. 1e and Extended Data Figs. 1b and 2a−c), and we found the same decrease in *T*_onset_ and increase in *A*_CR_ and peak velocity. Notably, the AUC exhibits an insignificant rise for this group of subjects, which suggests that the late kinematics of the CR may require more training to fully crystallize.

To distinguish between the possibility that the full probability distribution is being learned versus only the first time interval, we performed a control experiment that shows, indeed, that the full distribution influences the shape of the conditioned eyeblink response. In a control experiment, we introduced the Short prior condition, where the airpuff consistently follows the conditioned stimulus after a short duration, 200 ms, on every paired trial (100% chance on paired trials versus 20% on the Wide). Comparing metrics between these priors revealed marked differences in the resulting CRs (Fig. 1f). Despite reaching the same *A*_CR_, and sharing *T*_onset_, mice in the Short condition produced blinks with higher peak velocity and also reopened their eyes faster, resulting in a lower AUC and a narrower CR overall (Fig. 1f).

**Extended Data Fig. 3:**
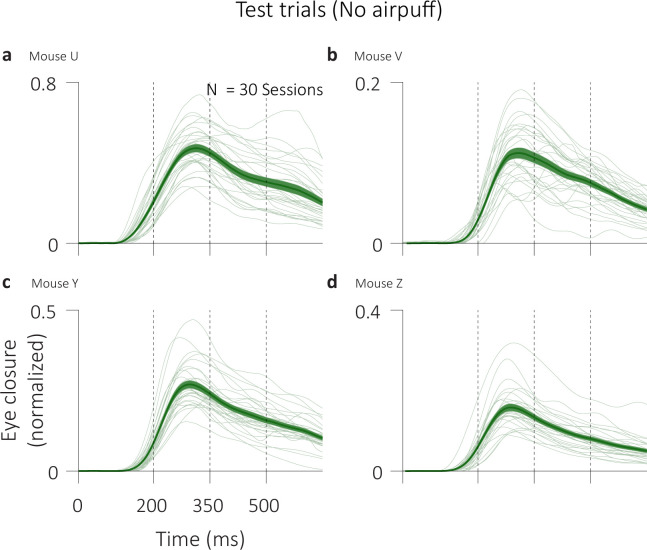
Examples of predictive eyelid responses of mice trained with the Bimodal prior. **a–d**) The average eyelid trace on test trials (no airpuff, predictive response) is shown for N = 30 sessions (thin lines, green) and the overall averages are shown (thick green line). Shaded region represents standard error of mean. Sessions were selected from the final stages of training and inclusion was based on mouse performance.

These findings suggest that, whereas certain features of the response, such as *T*_onset_ and *A*_CR_, may be more closely tied to the absolute timing of the stimulus, others, such as velocity and sustained closure (AUC), are modulated by the probabilistic context. Further support for this interpretation comes from mice trained under a Bimodal prior condition, characterized by a non-uniform temporal probability distribution with two distinct peaks (Fig. 1b and Extended Data Figs. 1a and 3a–d). In this condition, predictive eyeblinks exhibited a bimodal structure, often featuring two peaks in the average conditioned eyelid trace corresponding to the higher-probability intervals, demonstrating further that the full probability distribution is taken into account.

**Extended Data Fig. 4:**
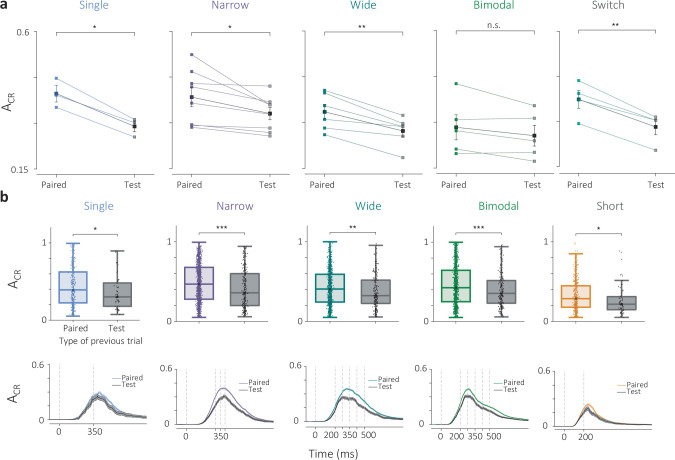
Analysis of the effect of previous trial on A_CR_. **a**) Amplitude of the conditioned response (A_CR_) on the N^th^ trial per mouse, contingent upon whether the previous trial (N-1^th^) was a paired or a test trial, where the airpuff was omitted for the Single (blue, t(2) = 7.68, p = 0.01), Narrow (purple, t(7) = 2.77), p = 0.02), Wide (cyan, t(5) = 4.97, p < 0.01), Bimodal (green, t(4) = 1.88, p = 0.13), and Switch (t(3) = 8.3, p < 0.01). Colored squares indicate individual median responses, and black squares represent averages. Error bars are standard error. **b**) Top: Examples of within individual effect of the N-1th trial. A_CR_ on test trials when the preceding trial was paired or test for Single (blue, t(397) = 2.27, p = 0.02), Narrow (purple, t(1556) = 4.38, p < 0.001), Wide (cyan, t(1086) = 3.12, p < 0.01), Bimodal (green, t(1304 = 3.48, p < 0.001)) and Short conditions (brown, t(424) = 2.56, p = 0.01). Whiskers represent confidence intervals. Below: Median A_CR_ traces for the examples shown above for the different conditions, where responses on trials where the preceding trials are shown in color and where the preceding trial was a test trial are shown in black. Shaded bars represent standard error.

Previous work showed that cerebellar-dependent behaviors are sensitive to trial-by-trial effects^43,44^, and, for eyeblink conditioning in particular, the *A*_CR_ values on test trials that follow a previous test trial were found to be attenuated compared to those that follow a paired trial^45^. We replicated the latter finding in our data (Extended Data Fig. 4a,b; statistics in Supplementary Table 1). However, we could not find any interval-specific trial-by-trial modulation in the conditioned eyeblink traces (statistics in Supplementary Table 2), suggesting that these traces may represent a consolidated response and not the average of diverse fluctuating responses.

Overall, these results establish that the predictive eyelid response reflects the uncertainty inherent in the full stimulus probability distribution.

### Cerebellar cortical activity changes concomitantly with temporal statistics and behavior

**Fig. 2:**
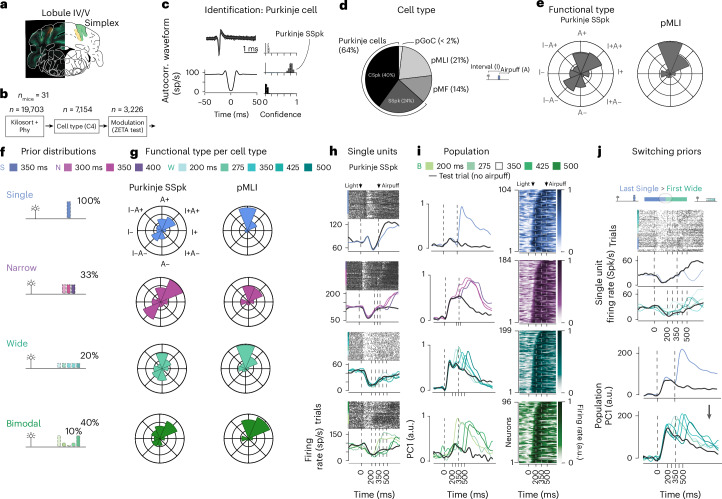
Cerebellar cortical activity encodes temporal statistics of prior distributions. **a**, Extracellular recordings were made from lobules IV/V and Simplex of the cerebellar cortex. **b**, Electrophysiological data (*n*_mice_ = 31) were obtained with Kilosort 2.0 and Phy^71^ (*n* = 19,703 units) and subsequently processed using C4 (ref. ^47^) (*n* = 7154 units), whereafter these units were checked for statistically significant task-related modulation using the ZETA method^48^ (*n* = 3,226 units). Only units that passed all these stages were included in the dataset. **c**, Example single unit labeled by the C4 method as Purkinje cell SSpks with waveform and autocorrelogram and showing high confidence score for Purkinje cell SSpks (middle) and low scores for pMLIs (top) and Purkinje cell CSpks (bottom). **d**, Summary statistics for cell type classification in the dataset. Sixty-four percent of task-modulating units are identified as Purkinje cells CSpk or SSpk. **e**, Functional classification of all statistically modulating Purkinje cell SSpks (left) and pMLIs (right) during the Interval (I, predictive component) and Airpuff (A, reflexive component) epochs, where + and − symbols represent statistically significant facilitation and suppression of activity in these epochs, respectively. **f**, Neural activity was recorded for the Single (S) (blue), Narrow (N) (purple), Wide (W) (cyan) and Bimodal (B) (green) prior distributions for paired trials (in color) and test trials (black). **g**, Similar to **e**, summary of functional classification Purkinje cell SSpks (left) and pMLIs (right) for each prior condition where Purkinje cells encode more interval-related information, whereas pMLIs encode largely airpuff-related sensory information. **h**, Examples of identified Purkinje cell SSpks of the same functional class for each prior condition. Recordings were made during both paired trials (with airpuff, in color) and test trials (without airpuff, in black). **i**, Left, examples of the first principal component (PC1) for populations recorded for different prior conditions across time (average variance explained by PC1: 43%). Right, summary of Purkinje cell SSpk population across all mice on test trials for different prior conditions for I+ functional classes. Dark colors represent large deviations from baseline; white represents activity at baseline. Onset time of activity and area under the modulation curve become earlier and larger, respectively, as the prior condition varies from Single to Wide. **j**, Switch mice. Top, example of a single putative Purkinje cell unit recorded as the subject switched from the last Single prior session to the first Wide prior session (Single > Wide), where a widening of modulation can be observed, especially in the test trial activity trace (black). Bottom, population summary (PC1) for a Single expert during the switch to Wide. Colored lines represent paired trials; black lines represent the test condition, when no airpuff was administered. Autocorr., autocorrelogram; sp/s, spikes per second; pGoC, putative Golgi cell; pMF, putative mossy fiber.

**Extended Data Fig. 5:**
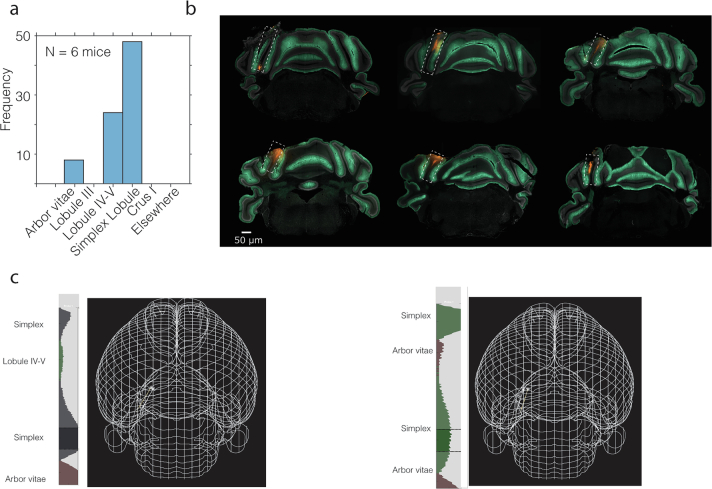
Quantification of recording sites in the cerebellar cortex. **a**) Histogram of locations of detected probe track points aligned to Allen Common coordinate framework (CCF). **b**) Examples of histological images used to identify probe entry locations as marked with DiI at grid point epicenter using a single shank on the last day of recording. The entry point consistently lies between lobules Simplex and IV/V. Recordings were made from ventral (max depth − 2200 μm) to dorsal locations (max depth 300 μm) with nonlinear double-shank electrodes. Note that perfusion was not performed on the same day after track-marking since animals continued to train on the Wide condition for several days for behavioral analysis. **c**) Allen CCF probe track analysis examples and probe tracks marked in a 3D mesh after registration with the Allen CCF framework for two mice.

**Extended Data Fig. 6:**
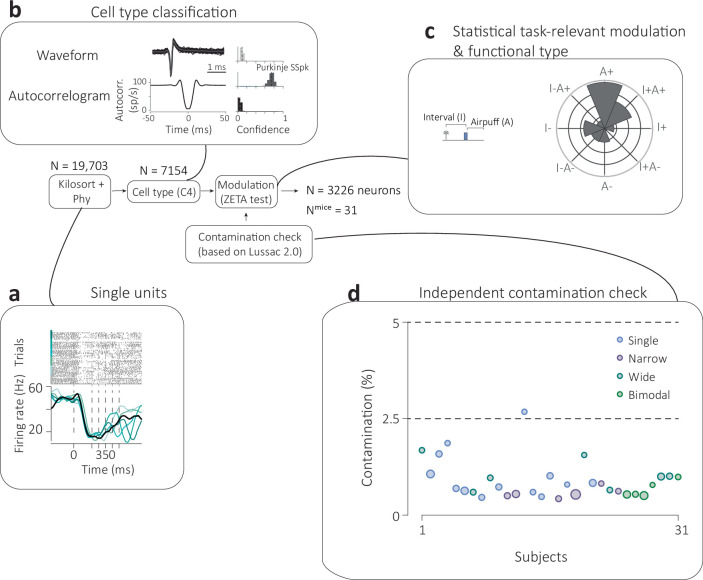
Unit selection pipeline for cell-type and task-related modulation with additional contamination evaluation. **a**) Recorded neural data was processed using Kilosort and curated in Phy^52^. **b**) These units were then passed into the C4 method^48^, which resulted in N = 7154 cell-type labelled single units. **c**) These units were tested for task-relevant statistical modulation using the ZETA method^49^, leading to N = 3226 single units. **d**) We then used specifications in Lussac^50^ to perform an independent contamination check of the single units to test quality and found the average contamination rate across mice (N = 31 mice) to be 0.8%.

**Extended Data Fig. 7:**
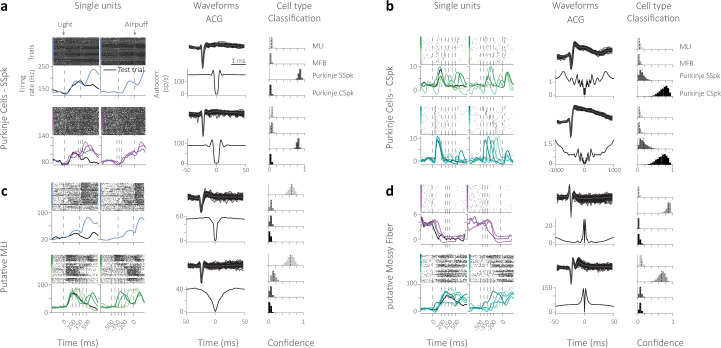
Identification of cell-types for all task-modulating units (N = 3226 units using C44^8^ and ZETA4^9^). **a**) Purkinje cell Simple Spike (SSpk) examples with rasters and firing rates (left) for the Single (blue: paired trials, black: test trials), Narrow (purple: paired trials, black: test trials), Wide (cyan: paired, black: test trials) and Bimodal (green: paired, black: test trials) conditions. Right: C4-identified waveforms, autocorrelograms and confidence of classification (rightmost column). **b**) Same as a) for Purkinje complex spikes (CSpk) for different prior conditions. **c**) Same as a) for putative MLIs (pMLI). **d**) Same as a) for putative Mossy Fibers (pMFB).

We next show that the activity profiles of cerebellar Purkinje cells reflect the statistics of the prior distribution and also resemble the predictive eyelid behavior (Fig. 2a−j). We performed acute large-scale electrophysiological recordings in lobules IV/V and Simplex of the mouse cerebellum, with probe locations confirmed by histological reconstruction and quantification after alignment to the Allen Brain Atlas^46^ (Fig. 2a and Extended Data Fig. 5a–c). Based on a pipeline to obtain single units using a combination of recent methodologies^47–49^ (Fig. 2b and Extended Data Fig. 6a–d), we identified neurons (*n* = 3,226 units; Fig. 2b–j) that are cell type identified (Fig. 2b–d,g and Extended Data Fig. 6a–d), show task-related modulation (Fig. 2b,e,h and Extended Data Figs. 6c and 7a–d) and exhibit low contamination (Extended Data Fig. 6d). Among these, we focused on Purkinje cell CSpks (40%; Fig. 2d), Purkinje cell SSpks (24%) and putative molecular layer interneurons (pMLIs, 21%).

Examining the task-dependent function of neural activity of different cell types, based on methods in previous work^44^, revealed that, whereas pMLIs were largely encoding information of the reflexive eyeblink component after the airpuff (Airpuff epoch; A+ and A− indicate positive or negative modulation), Purkinje cell SSpk activity encoded heterogeneous patterns of interval-related information pertaining to the predictive component (Interval epoch I; I+ and I− indicate modulation direction). This pattern held consistently across all prior conditions in the functional heterogeneity profiles of these cell types (Fig. 2e,g). Because the most relevant predictive information was consistently found in Purkinje cell activity, for the remainder of the paper we focus on these.

**Extended Data Fig. 8:**
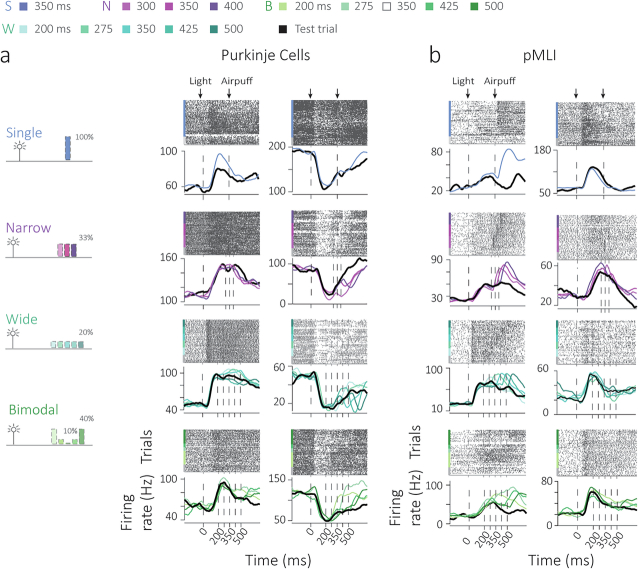
Examples of single units reflecting tuning that matches temporal statistics of prior. **a**) Purkinje cell rasters and firing rates over time, aligned to onset of the light (left panel) and airpuff (right panel) for the Single (paired trials: blue, test trials: black), Narrow (paired trials: purple, test trials: black), Wide (paired trials: cyan, test trials: black), and Bimodal (paired trials: green, test trials: black) conditions. **b**) Same as a) for putative molecular layer interneurons (pMLI) for the different prior conditions.

**Extended Data Fig. 9:**
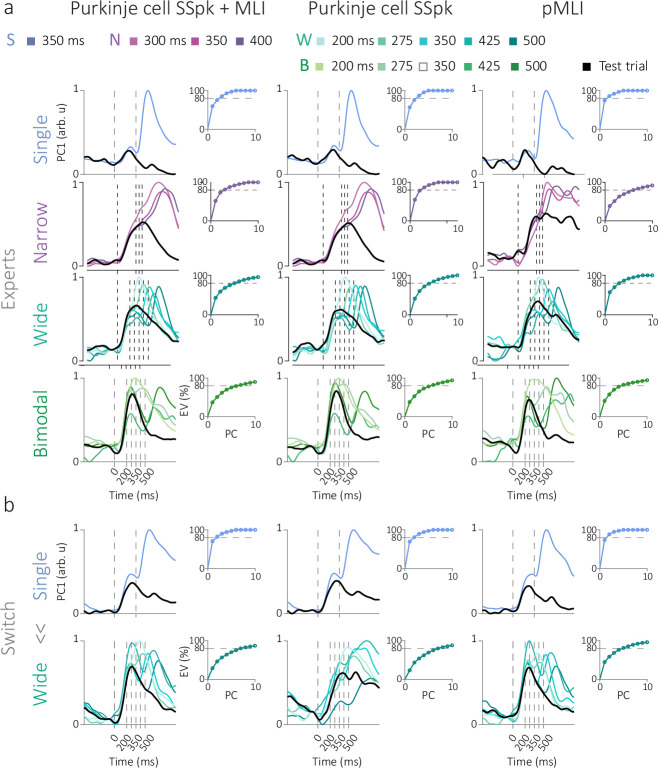
Principal components of cerebellar cortical neuron (Purkinje SSpk and pMLIs) and the relative population contributions of Purkinje SSpk and pMLIs. **a**) Left: Examples of cerebellar cortical populations (first principal component vs. time) from Single, Narrow, Wide, and Bimodal experts including on Purkinje SSpk and pMLI cell types. Black traces indicate projections of test trials and colored traces indicate projections of paired trials. Insets show Cumulative Percentage explained variance (EV%) with each principal component, showing that most are low-dimensional systems assuming an 80% threshold (N_dim_ « N_total_). Total number of neurons in each population is provided in Supplementary Table 9. Middle: Same as the left column but for each prior condition, the contribution of the Purkinje SSpk to the population. Right: Same as the left column but displays the contribution of the putative molecular layer interneurons (pMLIs) for the same mouse. **b**) Same as a) for switch mice where populations were recorded in the same mouse before and after the switch from Single to Wide.

Despite functional heterogeneity, a substantial fraction of Purkinje cell SSpks exhibited tuning profiles that closely tracked the first-order statistics of the prior distribution—specifically, the mean duration, standard deviation and overall width (Fig. 2i and Extended Data Fig. 8a,b). Principal component analysis (PCA) and visualizing the population activity of Purkinje cell SSpks for different prior conditions (Fig. 2i) revealed low-dimensional dynamics within cerebellar cortical populations (Extended Data Fig. 9a), with tuning properties that systematically broadened as the prior distribution widened (Fig. 2i, Extended Data Fig. 9a and Supplementary Table 3). Among the group of switch mice, this shift in tuning was evident both when individual Purkinje cells were recorded during transitions from the Single to Wide condition (Fig. 2j) and across populations as mice switched from Single to Wide conditions (Fig. 2j and Extended Data Fig. 9b). Thus, beyond consistent tuning changes across individual units in experts, we observed that Purkinje cell SSpk tuning broadened in alignment with the prior distribution in switch mice, mirroring changes at the population level.

**Fig. 3:**
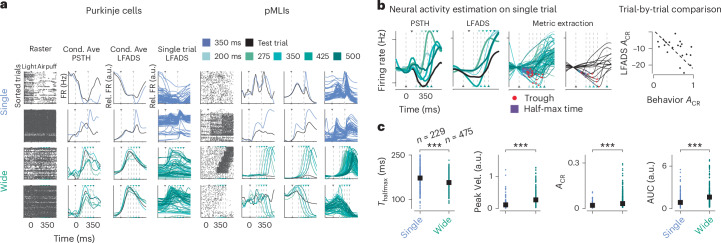
Relating Purkinje cell and pMLI activity to behavior and prior distributions on a trial-by-trial basis. **a**, LFADS^50^ was used to obtain trial-by-trial estimates of neural activity. Traces are shown for both paired trials (in color) and test trials (in black). Left to right: spike times sorted by conditions (raster), conditioned average firing rates (PSTH), conditioned averaged LFADS-inferred activity and LFADS inference of activity on individual trials in the Single condition—for example, Purkinje cells (left) and pMLIs (right) for switch mice during the Single (top, blue) and the Wide (bottom, cyan) conditions. **b**, Left, metrics extracted from LFADS-inferred trial-by-trial estimates of neural activity of switch mice recorded from Single and Wide sessions. Right, an example session showing trial-by-trial correlation between LFADS-derived *A*_CR_ and the behaviorally derived *A*_CR_ (*R* = −0.53, *P* < 0.05). **c**, LFADS-derived *t*-statistics of neural activity estimates for switch mice in the Single condition (*n* = 148 units) and the Wide condition (*n* = 342 units) recapitulate the pattern of changes observed in the behavioral results—that is, decrease in *T*_halfmax_ (##, *t*_702_ = −6.03, *P* < 0.0001) and increase in peak velocity (##, *t*_702_ = 6.68, *P* < 0.0001), *A*_CR_ (##, *t*_702_ = 7.34, *P* < 0.0001) and AUC (##, *t*_702_ = 5.17, *P* < 0.0001). Black squares represent averages; error bars represent standard error. ***P < 0.0005. Full statistics are provided in Supplementary Table 4. ## indicates two-sided *t*-test. Cond. Ave, conditioned average; Peak Vel., peak velocity.

**Extended Data Fig. 10:**
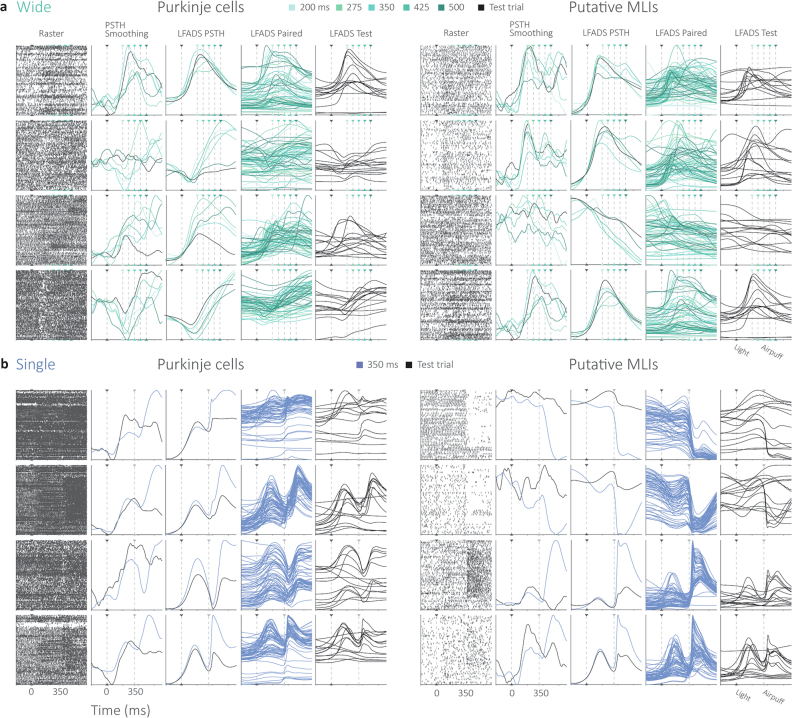
Examples of LFADS estimates of firing rates of Purkinje SSpk and pMLIs in switch mice. **a**) Left to right: Spike times organized trial-wise (raster), conditioned average firing rates, historically known as peri-stimulus time histograms or PSTH, conditioned averaged LFADS-inferred activity, LFADS inference of activity on individual trials in the Wide condition. Left: examples of Purkinje cells, Right: examples of putative molecular layer interneurons. **b**) Same as a) for the Single condition.

Although trial-averaged neural activity lacks the richness to enable direct comparison to behavior, by inferring trial-by-trial neural activity we were able to establish good correlation between metrics obtained from neural activity estimates and behaviorally derived metrics. More broadly, patterns of changes observed in metrics derived from neural activity matched the patterns observed in behavior (Fig. 1e). For this, we used a deep learning method called LFADS^50^, which infers latent initial conditions and dynamics from population spiking activity to estimate single-neuron firing rates. LFADS accurately reconstructed neural activity profiles for Purkinje cell SSpk units and pMLIs (Fig. 3a and Extended Data Fig. 10a,b). Trial-resolved neural metrics derived from LFADS outputs were significantly correlated with behavioral metrics (Fig. 3b and Supplementary Fig. 1a–c) and mirrored changes observed in *T*_onset_, peak velocity, AUC and *A*_CR_ (Fig. 3c; statistical results in Supplementary Table 4). Together, these results show that cerebellar cortical activity modulated systematically with the mouse’s behavior and the stimulus prior distribution on a trial-by-trial basis.

### A novel Purkinje cell CSpk signal encoding onset of prior distribution

**Fig. 4:**
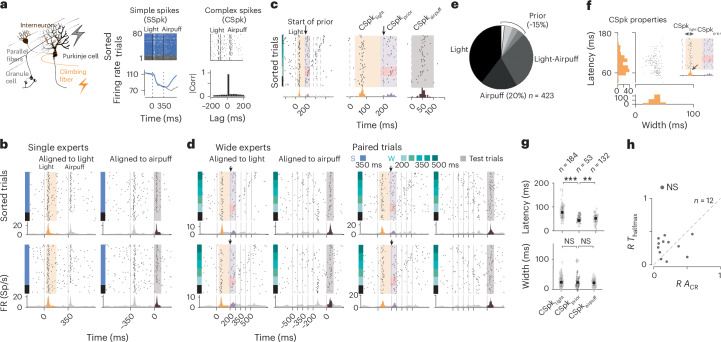
A CSpk signal that marks the onset of high-uncertainty prior distributions. **a**, Purkinje cells exhibit high-frequency SSpk activity and low-frequency CSpk activity (left, middle), which, when cross-correlated, reveal a 20-ms suppression of SSpks by CSpks (right bottom). **b**, In the Single condition, previous findings of a light-related CSpk (CSpk_light_, in orange) and an airpuff-related CSpk (CSpk_airpuff_, in brown) are recapitulated. **c**,**d**, In Wide experts, in addition to the CSpk_light_ and CSpk_airpuff_, a CSpk_prior_ is found (purple region counts toward the CSpk_prior_ histogram, and the red region is omitted), which is time-locked to the onset of the earliest probable interval of the Wide prior (in purple). **e**, Relative proportions of the three functional types of CSpks found in Wide experts. **f**, CSpk latency is computed as the interval (between event onset and spike time) at which the distribution peak of all individual spikes occurs. Width is computed as the time elapsed between the *T*_halfmax_ of the rise and decay phases of the instantaneous firing rate. **g**, The width and latency of CSpk_light,_ CSpk_airpuff_ and CSpk_prior_ are calculated at the time of the onset of the light, airpuff and first interval of the prior, respectively. *t*-statistics show the latency of CSpk_prior_ to be smaller than that of CSpk_light_ (##, *t*_449_ = 13.4, *P* < 0.001) and that of CSpk_airpuff_ (##, *t*_377_ = −2.94, *P*= 0.003). Box and whiskers indicate mean and s.e.m. *P < 0.05; **P < 0.01; ***P < 0.001. ## indicates two-sided *t*-test. **h**, Correlations of the time of the CSpk_prior_ with behavioral metrics *T*_halfmax_ and *A*_CR_. Non-significant (NS) correlations are shown in black. |Corr|, correlation amplitude; ~, approximately; FR, firing rate; Sp/s, spikes per second.

In addition to observing temporal statistics in the fast-spiking Purkinje cell SSpk presented in previous sections, we also discovered a novel prior-related Purkinje cell CSpk when the subject experienced prolonged exposure to high temporal uncertainty. Previous studies of classical delay eyeblink conditioning identified Purkinje cell CSpks after the presentation of a light cue (CSpk_light_)^29^ and after a periocular airpuff (CSpk_airpuff_)^51^. In line with these findings, we observed both functional CSpk types in our dataset across both the Single and Wide prior conditions: CSpk_light_ (*n*_Single_ = 231 units, *n*_Wide_ = 360) and CSpk_airpuff_ (*n*_Single_ = 289, *n*_Wide_ = 288) (Fig. 4a–d and Supplementary Fig. 2). These signals were identified using a recent method^48^, which we used to quantify their prevalence (Fig. 4e) and to characterize their temporal properties—specifically, latency and response width (defined in Fig. 4f and Supplementary Fig. 3a,b) relative to the onset of their associated sensory stimuli (that is, light and airpuff; Fig. 4g). However, in mice trained on the Wide condition, we encountered an unexpected CSpk signal in Purkinje cells that appeared to be coincident with the onset of the earliest probable interval of the prior distribution. We refer to this as the prior-related CSpk (CSpk_prior_; Fig. 4c,d and Supplementary Fig. 2). CSpk_prior_ comprised 15% of all functionally significant CSpks recorded in Wide experts (*n*_Wide_ = 91 Purkinje cell units; Fig. 4e). Unlike CSpk_light_ and CSpk_airpuff_, no external sensory event is tied to the vicinity of the CSpk_prior_ (the contaminating influence of CSpk_airpuff_ at 200 ms is excluded while calculating statistical modulation of CSpk_prior_). If we compute its latency from the onset of the earliest probable interval in the prior, we find this latency to be smaller than that obtained for CSpk_light_ and CSpk_airpuff_ relative to the light and airpuff, respectively (Fig. 4g; other statistics in Supplementary Table 5). The trial-by-trial time of onset of the CSpk_prior_ did not correlate with behavioral metrics of the eyeblink, such as *T*_onset_ or *A*_CR_ (Fig. 4h).

In a control experiment where we recorded Purkinje cell activity during the transition from the Single condition to the Wide condition (*n* = 30 Purkinje cell units; Supplementary Fig. 4a–d), we show that the CSpk_prior_ signal is not present at the outset. It may, therefore, represent a signal acquired through experience with longer exposure to the Wide prior condition. We did this for both naive groups of mice (*n*_mice_ = 5) and a subset of Single experts that would switch to the Wide prior (*n*_mice_ = 5). We found that, based on the statistical analysis presented earlier, the CSpk_airpuff_ spikes were present in naive and expert mice (*n*_neurons_ = 49 units; Fig. 4d,e and Supplementary Fig. 4c). On the other hand, the CSpk_light_ spikes for the switch group Purkinje cells were present mainly in the expert group (*n*_neurons_ = 12 units; Fig. 4b−e). The properties of CSpk_light_ and CSpk_airpuff_ did not change before and after the occurrence of the switch (Supplementary Fig. 4e; statistics in Supplementary Table 6). More importantly, we could not find the occurrence of any CSpk_prior_ in the naive mice, nor in the Single experts after they switched to the Wide condition (Fig. 4b and Supplementary Fig. 4d).

These results show that CSpk_prior_ does not appear to be present at the first instance that the cerebellar cortex encounters high-uncertainty temporal distributions but is prevalent in mice with prolonged exposure to high-uncertainty distributions, suggesting that it may intrinsically develop over time through plasticity in the olivary-cerebellar loop.

### Cerebellar cortical involvement in prior-related activity and behavior

**Fig. 5:**
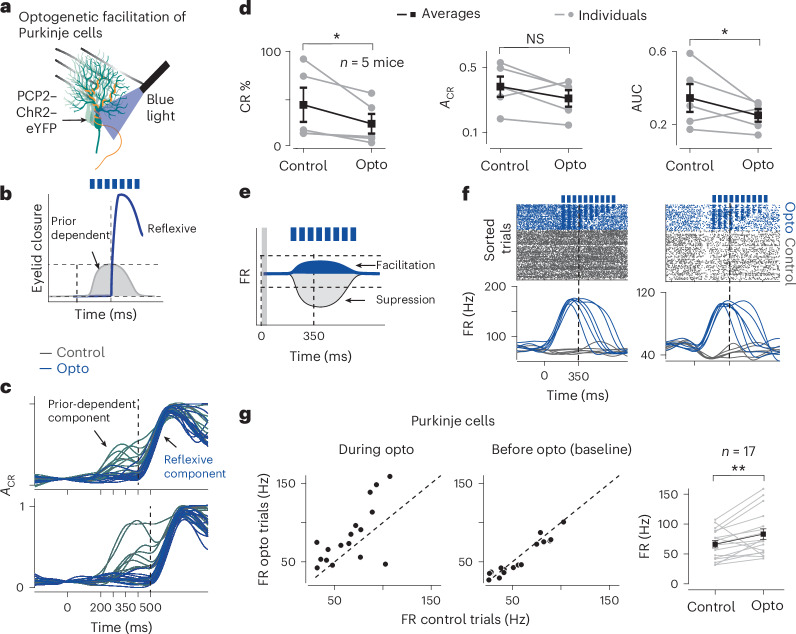
Optogenetic silencing of the prior-related predictive component of the eyeblink. **a**,**b**, Optogenetic strategy to probe the role of suppression of Purkinje cell activity during the predictive behavioral response: mutant mice expressing ChR2 in Purkinje cells (PCP2−Cre−ChR2−eYFP) were trained on the Wide prior condition. Electrophysiological recordings were performed as optogenetic perturbation with blue light was administered at random on 40% of trials within a session. **c**, Individual behavioral traces for different time intervals comparing trials with optogenetic perturbation (dark blue) versus controls (gray) within the same session on paired trials where, for normal conditions, both prior-dependent and reflexive components are expected to be present. **d**, Behavioral summary of *t*-statistics for CR percentage (#, *t*_4_ = −2.3, *P* = 0.04), *A*_CR_ (#, *t*_4_ = −1.98, *P* = 0.06) and AUC (#, *t*_4 _= −2.1, *P* = 0.05) comparing optogenetic and control trials within sessions of the same mouse. Black circles and lines indicate averages; gray circles and lines indicate individuals; error bars represent standard error. **e**, Precise optogenetics to wash out suppression dynamics during prior-related behavior. **f**, Rasters and firing rates of Purkinje cells during optogenetic sessions revealed the successful facilitation of these cells while remaining in the natural activity regime of Purkinje cells. **g**, Most Purkinje cells (*n* = 17) recorded exhibited a significant increase in firing rate during optogenetics trials compared to control trials (left) only during the optogenetic epoch and not during the baseline epoch (middle). Right, in general, Purkinje cells registered an increase in firing rate during optogenetic trials compared to that during control trials (#, *t*_16_ = 2.5, *P*= 0.01). Black lines and circles represent averages. Error bars are standard error. # indicates one-sided *t*-test. *P < 0.05; **P < 0.01. FR, firing rate; NS, not significant; opto, optogenetic.

We established the involvement of Purkinje cells in this probabilistic variant of trace eyeblink conditioning by eliciting a calibrated Purkinje cell facilitation during the prior distribution. This tests the hypothesis that it is the suppression of Purkinje cell SSpk activity that leads to the formation of the CR. In support of this, we found that such a perturbation reduces the occurence of the CR. Using an optogenetic strategy, we expressed Channelrhodopsin-2 (ChR2) in cerebellar Purkinje cells of mice (PCP2−Cre−ChR2−eYFP), implanted a tapered optic fiber in lobule simplex, simultaneously recorded cerebellar cortical neurons (Fig. 5a and Supplementary Fig. 5a,b) and stimulated at random on 40% of trials. We refer to the remaining trials as control trials. On optogenetic trials, we observed an elimination or severe attenuation of the predictive component of the eyeblink (Fig. 5b,c). In all cases, the reflexive component of the same motor behavior remained fully intact (Fig. 5c), which is thought to be influenced by a different cerebellar and reflex pathway^52^. Accordingly, when we compared the control and optogenetic trials, we found that the CR percentage and AUC significantly decreased (Fig. 5d; statistics in Supplementary Table 7). The firing rate of identified Purkinje cells was significantly higher during optogenetic stimulation than that during the baseline epoch of the same cell in the same session (Fig. 5e−g), showing no long-term disruption of neural activity. These results show that precisely timed facilitatory perturbation during the range of the prior in Simplex lobule Purkinje cells severely and reversibly impairs the prior-dependent component of the learned eyeblink response while leaving the reflexive component of the same behavior intact.

### An olivary-cerebellar mechanism for the acquisition of temporal statistics

**Fig. 6:**
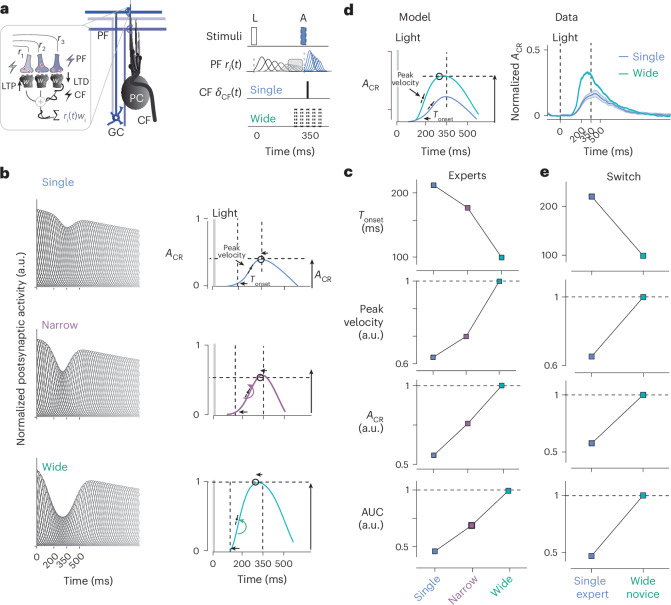
A cerebellar cortical mechanism for acquisition of temporal statistics. **a**, Cerebellar microcircuitry consisting of inputs to the Purkinje cell via GC axons and olivary climbing fibers (CFs). The model assumes that conjunctive activation of the GC and CF pathways leads to LTD, and activation of GCs alone leads to LTP of GC to Purkinje cell synapses. Purkinje cell activity above baseline is assumed to be a weighted sum of GC-related inputs. PF, parallel fiber. **b**, Model predictions for the postsynaptic activity after learning of temporal statistics. These results ensue from the gating of GC−Purkinje cell synaptic weights, leading to a decrease in net input activity that imprints the statistics of the temporal distribution. Right, averaging of these inputs and downstream linear combinations leads to model predictions for behavior for each prior condition. **c**, The decrease in *T*_onset_ and increase in peak velocity, *A*_CR_ and AUC are recapitulated for the Single (blue), Narrow (purple) and Wide (cyan) conditions. **d**, Model predictions and example of the behavior of a representative subject that switched from Single to Wide. **e**, The model recapitulates the behavior observed for behavioral patterns after Single to Wide switches. A, airpuff; L, light; PC, Purkinje cell.

We built a computational model of the cerebellar circuit to understand how the forces of LTD and LTP plasticity could interact during learning of probability distributions to give rise to prior-dependent memories in Purkinje cells. Our model uses previous proposals^53–58^ and extends them to provide a mechanism for encoding prior knowledge of temporal statistics. Previous proposals in cerebellar cortical theory focused on long-term plasticity mechanisms activated as a function of convergent sensory signals arising at the principal cerebellar cortical neuron: the Purkinje cell. During a trial, the model assumes that the light (conditioned stimulus) triggers a cascade of granule cell (GC) activations, giving rise to a decaying temporal basis (Fig. 6a), consistent with recent evidence^59–61^ and whose shape accommodates scalar variability in timing^58^. The computational model hypothesizes that each of these hundred thousand GCs makes synapses with Purkinje cells, funneling in potentially temporally heterogeneous but repeatable inputs time-locked to the onset of the light. After the interval elapses and the airpuff arrives, it activates the inferior olive, which elicits a CSpk in the Purkinje cell. The conjunctive activation of GCs and climbing fibers is thought to result in LTD of the subset of GC−Purkinje cell synapses active at a fixed time preceding the airpuff, which reflects the eligibility window for this lobule: 50 ms (but its actual value may be more varied^62^). In addition to this, the model employs an LTP mechanism in the absence of climbing fiber signals that seeks to return the system to homeostasis (Fig. 6a). The modeled rates of LTD and LTP are proportional to the net activity of GCs at different durations, which, given the temporal decay of the basis set, ensures that earlier airpuff-induced climbing fiber signals induce more pronounced LTD, and similar dynamics govern the rate of LTP albeit at a lower rate overall.

On top of that, the airpuff arrives at different times and with different probabilities in the probabilistic version of trace conditioning, influencing the model’s synaptic modifications through LTD (learning) or LTP (forgetting) at GC−Purkinje cell synapses in proportion to the probabilities of the climbing fiber input at different times, and, additionally, the model’s synaptic modifications are influenced by the occurrence of CSpk_prior_. This results in the selective gating of incoming GC inputs around the relevant intervals, whose synaptic weights mirror the probability of occurrence of the intervals of the distribution. Our modeling suggests that the prior-related CSpk additionally promotes LTD at the earliest intervals of high-uncertainty priors, resulting in larger *A*_CR_ for wide prior distributions (Fig. 6b). When these inputs are averaged over time, we found that behavioral predictions matched the increase in *A*_CR_, decrease in *T*_onset_ and increase in peak velocity and AUC, as observed in experts for the Single, Narrow and Wide priors (Fig. 6c). Furthermore, when we examined predictions for the switch mice as they transition from Single to Wide (Fig. 6d), we found the same decrease in *T*_onset_ and increase in peak velocity, *A*_CR_ and AUC (Fig. 6d,e).

## Discussion

Survival depends on detecting environmental regularities to predict future events. Through experience, organisms learn the prior probabilities of encountering specific stimuli. Bayesian theories propose that the brain internalizes these priors to guide behavior, a view supported by extensive work^1–6^. Here we provide one of the first reports that prior knowledge of the probabilistic statistics of stimuli shapes both neural activity and related behavioral outcomes concordantly. Our findings suggest that neural circuits imprint representations of probability in external stimuli as memories and use them to shape predictive motor outcomes under uncertainty.

Additionally, we also provide a concrete neural mechanism for how the juxtaposition of long-term plasticity rules and internal representations of time in cerebellar circuits can explain how probability distributions of stimuli with varying statistics can be internalized in Purkinje cell activity as prior knowledge. This computational model incorporates previous theoretical work^53,56–58,63^ to offer the insight that the same machinery is amenable to learning probability distributions. However, several observations and caveats have not been reconciled here. For instance, trace eyeblink conditioning has been attributed to other regions of the brain^64,65^ and it remains unclear what role cortico-cerebellar communication^34,66,67^ plays in supporting such behaviors. Furthermore, the model adopts a limited view of long-term plasticity in Purkinje cells, whereas a diversity of plasticity mechanisms in the cerebellum remain unaddressed^62,68^. Moreover, recent discoveries point toward novel population-level frameworks that explain how coherent downstream responses are driven^68^, which all suggest that further exploration of existing frameworks of cerebellar computations is needed.

One key finding we report here is that cerebellar Purkinje cells encode prior probabilities not only through their SSpk signaling but, over time, also develop CSpk signals that mark the onset of uncertain prior distributions. This signal does not appear to correlate with sensory or motor events. We hypothesize that this prior-related CSpk is an intrinsically generated prediction signal that mitigates uncertainty in the stimuli at the earliest intervals by enabling robust learning of the predictive eyeblink through LTD even when the stimulus does not occur often. This may explain why the amplitude of the predictive eyeblink response is larger for the distribution conditions not merely despite the uncertainty but also potentially because of it. Previous reports^29^ of CSpk_light_ propose the temporal difference prediction error theory, which would predict the suppression of CSpk signaling in the expected range of the prior distribution. However, here we found that, at the earliest probable interval, a CSpk_prior_reliably occurs, indicating the presence of distinct prediction mechanisms within the olivary-cerebellar system, warranting further theoretical investigation.

This finding may also be related to previous work that uncovered cerebral cortical neurons that begin to modulate in response to the onset of the stimulus distributions^15^. These areas are disynaptically connected with the cerebellum, and one could hypothesize that the olivary-cerebellar system may generate a predictive signal marking the onset of high-uncertainty distributions that may be used by downstream cortical areas to carry out complex behaviors.

Previous reports in human behavior indicate that humans found it difficult to accurately learn complex priors with multimodality or kurtosis, and behavior for only simpler priors is likely to be consistent with Bayesian inference models^19^. Our results and previous work^69^ suggest that eyeblink behavior in rodents and models of cerebellar cortical circuits may explain this observation. The nature of the decaying GC basis set hypothesized in our model and its interaction with Purkinje cell synapses may lead to an inadvertent low-pass filtering that may disadvantage the learning of temporal priors with multimodality, high kurtosis or other higher-order statistics. Evidence for such a basis set is increasingly common in literature^59–61^, and it remains to be seen how GC inputs are shaped by demands placed by different behaviors and neural computations.

In previous studies, cerebellar learning was characterized as occurring through summation of small trial-by-trial increments^45^. Some aspects of our findings reflect this phenomenon but not others. For instance, we report evidence of single-trial extinction on test trials that is expected from such a system, but we did not find further single-trial learning effects associated with the increased number of trial types introduced in the probabilistic version of trace conditioning. Nevertheless, it should be noted that considerable variation of CR traces was observed between behavioral trials in many conditions. We think that further study of these phenomena may yield more insight into intrinsic uncertainty and the stabilization of learning in cerebellar circuits and behaviors.

Overall, here we argue that the brain has evolved in environments where information is uncertain and probabilistic in nature. We demonstrate that neural circuits have the capacity to learn the probability distributions of task-relevant variables in the world and use these to shape implicit predictive behaviors—a premise long held by Bayesians in the behavioral, evolutionary and neuroscientific communities^70^. We hypothesize that exploring probabilistic behaviors may reveal new facets of neural mechanisms that optimize predictions under uncertainty.

## Methods

### Mice and surgical setup

This study used 48 mice (postnatal age >60 days, 21 females, from institutional colony). C57BL/6 mice were trained as experts in groups for five prior conditions: Single (*n* = 16 mice), Narrow (*n* = 9 mice), Wide (*n* = 9 mice), Short (*n* = 4 mice) and Bimodal (*n* = 5 mice). A subset of Single experts that transition to the Wide condition are called ‘switch’ mice (*n* = 15). Naive mice (*n* = 5) were also used as control subjects for electrophysiological recordings. Six L7-*cre* (BAC−*Pcp2*−IRES−*cre*^72^) mice crossed to Ai32 mice (*Rosa26*−LSL−*ChR2*−*eYFP*^73^) (The Jackson Laboratory) were trained on the Wide condition; of these, five mice were used for optogenetics (*n* = 5). All procedures were performed in accordance with protocols approved by the animal care and use committees at Erasmus Medical Center (protocol numbers sp2400103 and sp2100228). Mice were housed in a 12-hour light/dark cycle and were tested in the light phase. Ambient temperature was 25 °C at humidity levels of 40−60%. No restriction was placed on food. Water dispensation was maintained throughout, and body weight was monitored. All surgical procedures were carried out aseptically under a mixture of 3% isoflurane in 1 l min^−1^ oxygen anesthesia. Postoperative analgesia management was enabled by administering buprenorphine HCl (0.1 mg kg^−1^) and carprofen (5 mg kg^−1^). Mice were monitored and treated for 3 days after surgery before the continuation of experiments. Between postnatal age P60 and P80, a semimagnetized pedestal was installed on the skull to enable head fixation during behavioral training. After stabilization of behavioral metrics on the Single condition (Extended Data Fig. 2c), a custom-designed thermoplastic recording chamber was installed on a cerebellar craniotomy centered at AP −6.25 mm and ML −2.25 mm from bregma and 3 mm in diameter. The chamber was affixed using an adhesive (OptiBond; Kerr Corporation) and dental cement (Charisma; Kulzer). After implantation, the chamber of each mouse was cleaned daily with saline and a dura-cleaning tool and disinfected with low concentrations of ethanol to maintain the hygiene of the dura and surroundings. All surgical installations on the skull were aligned using two-dimensional line level. During recording sessions, a custom-made Plexiglass grid was installed into the chamber to ensure systematic anatomical access to cerebellar structures and to provide stable housing for the electrode. In optogenetics experiments, the tapered optic fiber (Sigma fiber; OptogeniX) was implanted through an adjacent grid location at an angle of 7°.

### Task, experimental setup, design and training

Mice were head-fixed to a post with a semimagnetized pedestal attached to their skull and were able to comfortably rest or move freely on a self-initiating treadmill with low forward resistance but adequate textured grip. A high-speed infrared camera (ace acA1300; Basler) was trained on the eye of the animal to record movements. Our setup was designed based on previous proposals for similar tasks^74^. Posterior whiskers were trimmed to minimize interference with eyeblink detection. A custom-made device delivered a periocular airpuff representing the unconditioned stimulus using an air-pressurized drive triggered by a 5-V pulse. A semicircular array of white LEDs was used to deliver the light representing the conditioned stimulus. This delivery system was built to ensure homogenous and adequate bilateral visual input to the mouse (to ensure equivalent bilateral activation of the pontine nuclei). The camera recording and stimulus delivery system were integrated using custom drivers and code in Objective C (Cocoa framework XCode; Apple) and MATLAB 2020a (MathWorks). Pulse Pal (Sanworks) was used for regulating stimulus delivery.

Each experimental training session lasted for 80 trials, with an intertrial interval sampled from a discretized truncated exponential function (*τ* = 4 s). Mice were given an expert status when the CR percentage values saturated to or exceeded a threshold of approximately 40% (Extended Data Fig. 2c). After performance metrics stabilized, mice underwent a surgical craniotomy and chamber placement. After recovery, expert mice continued training on the same condition. For Single experts, after several electrophysiological recording sessions with the Single condition, behavior was switched to the Wide condition. After discontinuing electrophysiological recordings, the mouse continued with behavioral training on the Wide condition (Extended Data Fig. 2c).

On each trial, the LEDs (light) were active for 70 ms, followed by the interstimulus interval (ISI), after which the airpuff was administered for 70 ms, resulting in reflexive eye closure. The ISI was determined based on the prior condition. For the Single prior, the ISI was 350 ms; for the Narrow prior, the ISI was uniformly sampled from a discrete distribution: [300, 350, 400] ms. For the Wide prior, it was also sampled with uniform probability from a discrete distribution: [200, 275, 350, 425, 500] ms. The Short prior used an ISI of 200 ms. In the Bimodal prior, there was a 40% probability of sampling the first and fifth interval and a 10% probability of sampling the second and fourth interval from the distribution [200, 275, 350, 425, 500]. Sessions of all conditions contained randomly interspersed test trials where the airpuff was omitted; these trials did not influence the probability calculations on paired trials. At the start of each session, two airpuff-only trials were used to compute and calibrate the baseline eye closure by calculating the change in pixel value during each frame in the region of interest. This value was used to normalize subsequent eyeblink responses. Mice were monitored at all times during training and were given a timeout if squinting or extended eye closure was detected.

### Quantification of eyeblink metrics

Eyeblink responses within the session were normalized to the reflexive response to the airpuff, which was recorded in absence of the light stimulus on airpuff-only trials (administered just before the start of the session) and on paired trials. A CR was detected if the eyeblink trace velocity increased beyond threshold and the amplitude exceeded three times the baseline standard deviation before the light presentation across all trials within a session. (1) CR percentage was computed as the percentage of trials where a CR was detected against the total number of trials within the session. (2) *A*_CR_ was computed as the maximum eye closure of the predictive component after light onset. (3) *T*_onset_ was computed as the time when the derivative of the eyelid trace first exceeded a speed threshold. (4) AUC was computed as the sum of the eyeblink trace on test trials within 600 ms of light presentation. (5) The peak velocity of the initial rise was considered as the highest velocity registered before peak eyelid closure is achieved. The (6) amplitude and (7) onset time at halfmax (*T*_halfmax_) were computed at half the CR amplitude. Responses were computed per session, and, for averages across sessions, only sessions where the CR% exceeded 40% were considered.

### Large-scale electrophysiology using silicon probes

Extracellular recordings were performed using ESSY-37 E1 32 channel silicon probes (Cambridge NeuroTech). An Intan (RHD2132) amplifier was used to digitize and amplify the recorded extracellular voltage signals at 16 bits, which were recorded using an Intan RHD2000 Amplifier Evaluation System (sampling rate: 30,000 Hz). We used Open Ephys^75^ for acquisition, online monitoring and processing of cerebellar electrophysiological signals. A craniotomy 3 mm in diameter was made at AP −6.25 mm and ML −2.25 mm, after which a cylindrical lightweight recording chamber with a sealable lid was installed at the rim of the craniotomy on the skull surface. For a subset of the mice, a Plexiglass grid, designed to fit into the chamber at a horizontal orientation, was assembled, and the silicon probes were lowered through the grid holes. Our quantification of histological results shows that we recorded from lobules IV/V and simplex lobules consistently. On average, 13.4 sessions (s.d. = 8.5) per mouse were recorded at different depths unidirectionally from ventral to dorsal. The silicon probe was allowed to stabilize for 20 minutes before recording. The mouse could move freely on the treadmill at all times without influencing probe stability.

### Optogenetics

We implanted the tapered optogenetic fiber (Sigma fiber; OptogeniX) at a 7° angle from an adjacent grid hole to cover Purkinje cells recorded from lobule V from a depth of 1,125−2,000 µm on the ventrolateral aspect. We used mice that expressed ChR2 in Purkinje cells (PCP2−Cre−ChR2−eYFP), where uniformity of expression was confirmed post hoc through histological analysis of the co-expressing eYFP marker. The power of the LED source was calibrated over pilot experiments to 0.5 mW, which allowed the majority of Purkinje cells recorded to remain below 150 Hz, where the natural firing range of Purkinje cells is 40−200 Hz. Each optogenetics session consisted of 120 trials; on 40% of the trials, a variable pulse train of 20 Hz was delivered to cover the model-predicted time of suppression until after the airpuff. Rasters were sorted based on optogenetic stimulation condition and, thereafter, based on trial condition.

#### Single-unit isolation pipeline, cell type identification, modulation and contamination checks

Single-unit isolation using Kilosort and preliminary cell type screening

Neural recordings were analyzed for single units using Kilosort 2.0 (ref. ^71^) with default parameters, and units were curated manually using Phy, which resulted in *n* ≈ 19,000 putative single units were based on an tolerated contamination rate (defined as violations of refractory period in the interspike interval histogram) of 5%. Preliminary screenings of putative Purkinje cells were performed on five criteria. (1) Recordings were performed from the molecular layer and the Purkinje layer based on the polarities of the identified complex spike patterns. (2) The baseline firing rate of neurons lies between 40 Hz and 200 Hz. (3) CSpk and SSpk waveforms were recorded from the same channel or adjacent locations within 20 μm. (4) The CSpk and SSpk waveforms conformed to standard timescale and shape properties^76^. (5) The CSpk elicited a 20-ms SSpk suppression in a cross-correlogram.

#### Cell type identification using C4

These data, after being processed by Kilosort and Phy, were then additionally processed using the C4 method^47^, which uses waveform and discharge statistics in a deep learning framework to estimate cerebellar cell types (Extended Data Fig. 7a–d). For C4, we used a confidence ratio threshold of 1.5, and high-confidence Purkinje cells were cross-checked with prescreened units. Other cell types that were labeled as MLIs, Golgi cells and mossy fibers were identified as putative units for each of these cell types. For the experiments in Supplementary Fig. 2, where the same units were recorded during the switch from the last Single to the first Wide condition, given the challenging nature of the experiments and limited units available per mouse, we do not always use C4-identified units to examine units, and these units may contain higher contamination compared to the remainder of the dataset.

#### Functional classification of cerebellar cortical population

Modulatory activity was detected using a recent statistical method known as the ZETA test^48^. For Purkinje cell simple spiking and pMLI activity, the test was applied separately to spikes in the paired and test conditions for detecting modulation with respect to the airpuff and the light, respectively. For those neurons showing significant modulations, polarities were determined by comparing baseline and within-trial spiking activity. Baseline spiking was always computed in the −150-ms to −50-ms window relative to the light. Within-trial spiking for light-modulated activity was computed in test trials in the 100−500-ms window after the light. Within-trial spiking for airpuff-modulated activity was computed in airpuff-aligned rasters in the 50−200-ms window after the airpuff. For Purkinje cell CSpks, modulatory activity was detected by applying the ZETA test to windows surrounding events of interest: Light, Prior-onset and Airpuff. CSpk_light_ was detected in the 0−150-ms window after the light, and CSpk_prior_ was detected in the 190−275-ms window after the light or, equivalently, the −10-ms to 75-ms window surrounding the onset of the prior in the Wide condition. While computing the firing rates and significant modulation for CSpk_prior_, we did not take the 200-ms condition into consideration due to the possibility of contamination with CSpk_airpuff_. Note that the precision of CSpk_prior_ is much smaller than the full window length. CSpk_airpuff_ was detected in airpuff-aligned rasters in the 0−150-ms window after the airpuff.

#### Contamination check using Lussac

To independently check single-unit quality of our electrophysiology pipeline, we use methodology from Lussac^49^. Based on this, we defined a ‘censored’ window and a refractory window. The censored window serves to exclude potential duplicate detection of single spikes from contamination estimates, and the refractory window determines the spikes that count toward the contamination. Based on the cell type labels for the single units obtained from C4, we used censored window spans of 1.5 ms and refractory window spans of 3 ms for Purkinje cell CSpks; for all other cell types and for Purkinje cell SSpks, we used censored window spans of 0.2−0.3 ms and refractory window spans of 1 ms.

### Analysis of Purkinje cell and pMLI populations

We used PCA on all C4 modulating units (that is, after ZETA test) to characterize how the neural population dynamics evolved in each prior condition (Single, Wide, Narrow and Bimodal expert conditions and switch mice (Single > Wide) conditions) as a function of time (Fig. 2j and Extended Data Fig. 9a,b). To evaluate the contribution of different cell types, we also performed PCA separately on Purkinje cells and on pMLIs (Extended Data Fig. 9a) and found that dynamics were largely preserved across cell types.

For the switch mice, although PCA was applied separately before and after the switch, mice were selected only if there was a similar number of neurons recorded in both conditions. PCA was then performed on a covariance matrix comprising the collective demeaned firing rates of cerebellar cortical populations (*n* neurons) over time bins *t*, within mouse and within condition *c*, without attrition and with an approximately equal number of trials.

1 $${C}_{{ij}}=\frac{1}{{TC}}\mathop{\sum }\limits_{t={t}_{0}}^{{t}_{f}}\mathop{\sum }\limits_{c=1}^{C}({r}_{c}^{i}(t)-\overline{{r}_{i}})({r}_{c}^{j}(t)-\overline{{r}_{j}})$$

Here, *C*_*ij*_ represents the covariance matrix between neurons *i* and *j*, with *T* representing the total number of time bins between *t*_*f*_ and *t*_0_ and *C* the number of conditions within each prior. ${r}_{c}^{i}(t)$ is the average firing rate of neuron *i* under condition *c* at time *t*, and ${\overline{{r}^{i}}}_{c}$ is the mean firing rate of neuron *i* across all time bins, with analogous notation for neuron *j*. The diagonalization of the covariance matrix *C* = *UDU*^T^, yields a new coordinate system given by the columns of the matrix *U*. *D* is a diagonal matrix representing the eigenvalues of the system, indicating the variance captured by the corresponding principal components (PCs). The columns of *U* correspond to the largest eigenvalues for the eigenvectors that explain the most variance in the neural data and are taken in descending order to be the PCs of the system. The projection of the *n*-dimensional data onto the *k*^th^ PC is given by:

2 $${\mathrm{PC}}_{k}\left(t,c\right)=\mathop{\sum }\limits_{i=1}^{N}{U}_{{ki}}\left({r}_{c}^{i}\left(t\right)-\overline{{{r}^{i}}_{c}}\right)$$

where ${U}_{k}^{i}$ is the *i*^th^ element of the *k*^th^ PC (*U*_*k*_). The PCs are linear readouts of the population activity, and the contribution of each neuron to a given ${\mathrm{PC}}_{k}\left(t,c\right)$ is given by the *i*^th^ element of *U*_*k*_. The first PC accounted for 43% variance on average across mice (further details in Supplementary Table 3), whereas the second PC often accounted for less than 10% variance for most subjects (further details in Supplementary Table 3). We performed a scree analysis to determine the dimensionality for which 80% of the variance could be explained. This number was around 3 or 4 for most mice and conditions. Geometric analysis was, therefore, performed on test trial projections of the neural population for the first three PCs.

### Trial-by-trial analysis of neural activity using LFADS

We used the LFADS framework^50^ to infer latent dynamics from the spike-sorted data recorded in switch mice during the Single and Wide conditions from cerebellar lobule simplex. After successful training runs, LFADS produced a smooth trial-by-trial firing rate estimate for each neuron. We used LFADS in its ‘multi-session stitching’ mode, which enabled the assimilation of multiple datasets originating from the same generative dynamics to be combined in a single neural activity model. We thus combined datasets across sessions but not across prior conditions or mice.

A standard cross-validation procedure was followed where a subset of the dataset was left out during training. The cost function was specified to minimize reconstruction cost and regularize the network (KL and L2). Generalizability was assessed through the test error. We lowered the starting weight for the KL term before training to prioritize regularization by other means. We also inspected all smooth firing rate inferences and compared those side by side with the corresponding spike rasters and peristimulus time histograms (PSTHs). We terminated training of networks based on asymptotic saturation of learning rate and test error. In addition to using LFADS tools provided in Pandarinath et al.^50^, further custom processing of LFADS was performed. Metric extraction on LFADS-inferred traces was performed after removing the mean initial condition variations from each trial. A summary of hyperparameters used for the LFADS models can be found in Supplementary Table 8.

### Encoding model for time intervals and scalar variability

We assume that GC spike counts (*r*) obey an inhomogeneous Poisson process whose rate function is Gaussian with mean *ω*(*t*) and standard deviation *σ*_*i*_. The maximum firing rate of the *i*^th^ GC parallel fiber, *μ*_*i*_, is specified with respect to the onset of the light or conditioned stimulus.

3 $$p(r,|,t)=\prod \displaystyle \frac{1}{{r}_{i}!}{\omega }_{i}{(t)}^{{r}_{i}}{e}^{-{\omega }_{i}(t)}$$

4 $${\omega }_{i}(t)=\displaystyle \frac{1}{\sqrt{2\pi {\sigma }_{i}^{2}}}{e}^{-\displaystyle \frac{{(t-{\mu }_{i})}^{2}}{2{\sigma }_{i}^{2}}}$$

Due to scalar variability, the internal estimate of elapsed time ($\widetilde{t}$) has a probabilistic relationship to the chronometric elapsed time (*t*). We formulated this relationship as a conditional Gaussian probability distribution whose mean is $t$ and whose standard deviation remains constant for the ${i}^{\mathrm{th}}$ kernel but scales across kernels by linear scaling factor ${w}_{b}$, equivalent to the Weber fraction that best describes behavioral observations. Therefore, we assume a heterogeneous population that takes such a form and approximates Weber’s law.

5 $$p(\tilde{t},|,t)=\displaystyle \frac{1}{\sqrt{2\pi {({w}_{b}t)}_{i}^{2}}}{e}^{-\displaystyle \frac{{(\tilde{t}-t)}^{2}}{2{({w}_{b}t)}_{i}^{2}}}$$

We will now assume a relatively dense and discrete heterogeneous population over stimulus time *t*_*s*_. Let *p*(*t*_*s*_) be the prior probability of the stimulus time *t*_*s*_. Although each GC may have a preferred firing time, only a subset of GCs will be active (over elapsed time) when a given *t*_*s*_ is administered. Previous work^77^ provided closed-form solutions for infomax allocation of tuning curves as a function of sensory prior knowledge when using a heterogeneous basis set if parameterized as follows:

6 $${h}_{i}\left({t}_{s}\right)=\phi h\left(d\left({t}_{s}-n/d\right)\right)$$

where *ϕ* is a gain term that modulates the maximum average firing rate of each neuron in the population, and density *d* controls spacing and width of tuning curves. However, in our case, given the large value of *n* compared to the timescale of a task, the density term has a lesser impact than in the sensory case described by Ganguli and Simoncelli^77^. On the other hand, if *n* is too large, the basis set may cease to behave as a low-pass filter. Therefore, there is an optimal value of *n* for which the model best describes observations in the high noise regime. We assume that, for a population of *n* neurons, there is a maximum firing rate *R* that provides an upper bound over the prior and full population:

7 $$\int p\left({t}_{s}\right)\mathop{\sum }\limits_{i=1}^{N}{h}_{i}\left({t}_{s}\right)d{t}_{s}\le R$$

Given these formulations, the Fisher information *I*_*f*_ (*t*) can be written as:

8 $${I}_{f}\left({t}_{s}\right)={d}^{2}\phi \mathop{\sum }\limits_{n=1}^{N\left(d\right)}\phi \left(d{t}_{s}-{t}_{n}\right)$$

9 $$\approx {d}^{2}\phi \sum \frac{{h}^{2{\prime} }\left({t}_{s}-{t}_{n}\right)}{h\left({t}_{s}-{t}_{n}\right)}$$

Given the anatomy of the circuit, *n* is large (we approximate it with 5,000 neurons), and we assume densely tiled firing rates based on recent experimental evidence in related tasks^61,78^. This implies that, here, such a large *n* is expected to result in a negligible change in density in the postsynaptic effect of the basis set after learning. In other words, after applying realistic anatomical constraints, we find that GC tuning may not have to rapidly shift to accommodate the prior. Therefore, the primary factor driving efficient allocation here is modulation to effective firing rates reflecting temporal priors.

Beyond the efficient coding parametrization, the tuning curves of our GC population before learning take a specific form to accommodate scalar variability. The firing rate reduction over the population was modeled by a gain function, *g*(*t*), with time constant *τ*_basis_, and the increase in width was modeled linearly before learning, ${{\sigma }_{\mathrm{basis}}}_{i}={\sigma }_{0}i\kappa /N$, where *i* indexes neurons ordered according to their preferred time interval; *N* is the total number of neurons; and *κ* is the proportion of increase in the width *σ*_0_. The resulting function describes the rate of the *i*^th^ GC:

10 $${r}_{i}(t)=g(t)\displaystyle \frac{1}{\sqrt{2\pi {\sigma }_{{\mathrm{basis}}_{i}}^{2}}}{e}^{-{(t-{t}_{s})}^{2}/2{\sigma }_{{\mathrm{basis}}_{i}}^{2}}$$

For encoding of *p*(*t*_*s*_), we define *w*_*i*_, which represents the postsynaptic weight of the *i*^th^ GC with a Purkinje cell. LTD in TRACE is modeled for each GC−Purkinje cell synapse as proportional to the rate of firing of respective GCs shortly before the firing of climbing fibers at the time of the airpuff. After learning the onset of the prior, the model also assumes a climbing fiber at the earliest interval of a prior distribution with non-trivial uncertainty. The time before climbing fiber firing at which GC−Purkinje cell synapses become eligible for LTD is called the eligibility trace ($\epsilon$), which we assume occurs 50 ms before the onset of the airpuff in the model^62^. In the absence of climbing fiber stimulation and in the presence of GC firing, a weak restoring force (LTP) acts to reverse learning. The dynamics of LTD and LTP were governed by their respective time constants, *τ*_*ltd*_ and *τ*_*ltp*_. In the absence of learning, synapses would gradually drift toward the baseline *w*_0_.

11 $$\frac{d{w}_{i}}{{dt}}=-\frac{1}{{\tau }_{{ltd}}}{r}_{i}\left({t}_{s}-\epsilon \right)\delta \left(t-{t}_{s}\right)+\frac{1}{{\tau }_{{ltp}}}\left({w}_{0}-{w}_{i}\right)$$

At steady state, the sum of all weights over the basis set population resembles the shape of an inverted prior distribution *p*(*t*), which makes sense given that Purkinje cells are inhibitory and one of the primary learning mechanisms in the cerebellar cortex is LTD^79^ of Purkinje cell activity. In the model, the change in baseline Purkinje cell activity is computed as a weighted sum of GC activity.

12 $$\Delta {V}_{{pc}}\left(t\right)=\sum {r}_{i}\left(t\right){w}_{i}$$

The presence of the eligibility trace implies that any climbing fiber firing at the onset of the light cue will have no bearing on the plasticity of the GC−Purkinje cell synapses. Similarly, GC activation at the time of the airpuff will be irrelevant to learning of the prior. Furthermore, our results remain qualitatively unchanged under assumptions of more complex functions of the eligibility trace.

### Statistics and reproducibility

No statistical method was used to predetermine sample size. Mice were excluded if they were unable to learn the task (after 1 month of training were unable to produce a conditioned eyelid closure in 40% of the trials) or if, due to surgical or implant failure, we were unable to continue experiments. The experiments were not randomized. The investigators were not blinded to allocation during experiments and outcome assessment.

### Reporting summary

Further information on research design is available in the Nature Portfolio Reporting Summary linked to this article.

## Online content

Any methods, additional references, Nature Portfolio reporting summaries, source data, extended data, supplementary information, acknowledgements, peer review information; details of author contributions and competing interests; and statements of data and code availability are available at 10.1038/s41593-026-02255-7.

## Supplementary information

Supplementary information Supplementary Figs. 1−5 and Supplementary Tables 1−9.

Reporting Summary

## Data Availability

Data generated in this study have been deposited in the Dryad database (10.5061/dryad.q573n5tz1).

## Code availability

The code developed for this study is provided on Zenodo^80^. Open-access code of methods that we used to different extents can be found here: (1) Kilosort version 2.0 and Phy version 2.0 (https://github.com/MouseLand/Kilosort/releases/tag/v2.0, https://github.com/cortex-lab/phy/ releases/tag/v2.0a1); (2) LFADS (https://lfads.github.io/lfads-run-manager/releases/tag/v0.3.1); (3) Zeta (https://github.com/JorritMontijn/zetatest/ releases/tag/v3.1.5); (4) C4 (https://github.com/m-beau/NeuroPyxels/tree/cell_types_classifier/tree/bcbae4ce26a91e1bb1f006954d763790eca65f72); (5) Lussac 2.0 (https://github.com/BarbourLab/lussac/releases/tag/v2.0rc5); and (6) Allen Common Coordinate Framework (https://github.com/cortex-lab/allenCCF/tree/9d21d8cd2fa982b959b26db528851d3bb9af57c1).
