# Supplementary material for: Neural circuits encode prior knowledge of temporal statistics
Source: Nat Neurosci. 2026 Apr 7;29(6):1452–61. doi: 10.1038/s41593-026-02255-7 (PMC13246449; doi:10.1038/s41593-026-02255-7)
Supplement: Supplementary file 1 — Supplementary Figs. 1−5 and Supplementary Tables 1−9. [file 41593_2026_2255_MOESM1_ESM.pdf]

nature neuroscience

Supplementary information https://doi.org/10.1038/s41593-026-02255-7

Neural circuits encode prior knowledge of temporal statistics

In the format provided by the authors and unedited

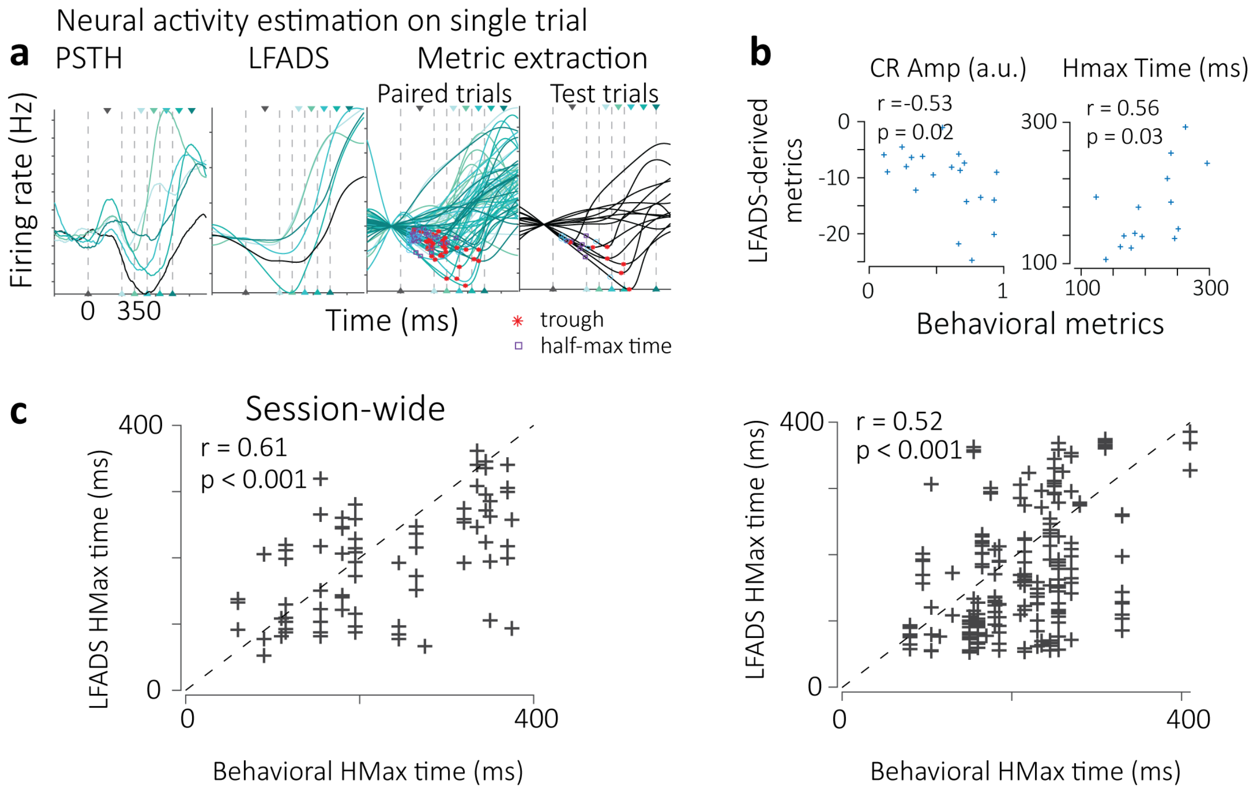

Supplementary Figure 1: LFADS trial-by-trial metric decoding. a) Comparison of condition-averaged firing rates of neural data vs. condition-averaged LFADS-estimates of firing rates and individual trialwise estimates for the paired and test conditions. The trough (minima, asterisk) and half-max time (square) estimates are indicated. b) Correlation of behaviorally extracted metrics of CR-amplitude and half-max time to LFADS estimates of firing rate trough and half-max time. c) Session-wide comparison between behavioral metrics and LFADS-derived metrics for half-max time.

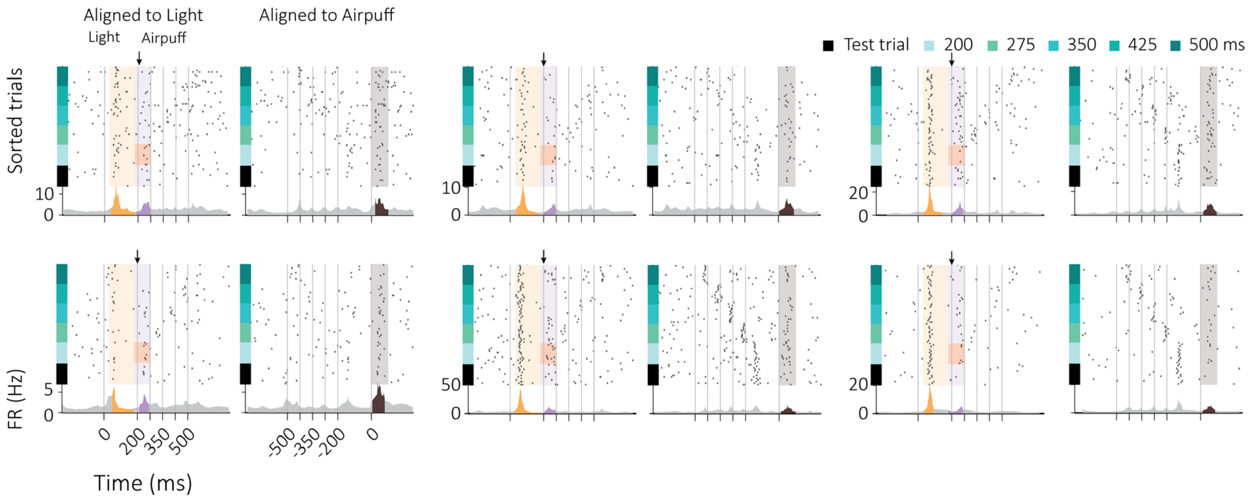

Supplementary Figure 2: Purkinje cell complex spikes in the Wide condition. a) In addition to $CSpk_{Light}$ (orange) and $CSpk_{Airpuff}$ (brown~~gray~~) signals, we additionally encounter a complex spike signal following the onset of the prior, $CSpk_{Prior}$ (purple region indicates contribution to histogram, red region is omitted). b) Top: Complex spike rasters aligned to the light (left panel) and airpuff (right panel). Bottom: Instantaneous firing rate estimates over time inferred from multiple timescales of spiking activity [49]. The light, prior, and airpuff signals are shown in orange, purple, and brown~~gray~~, respectively.

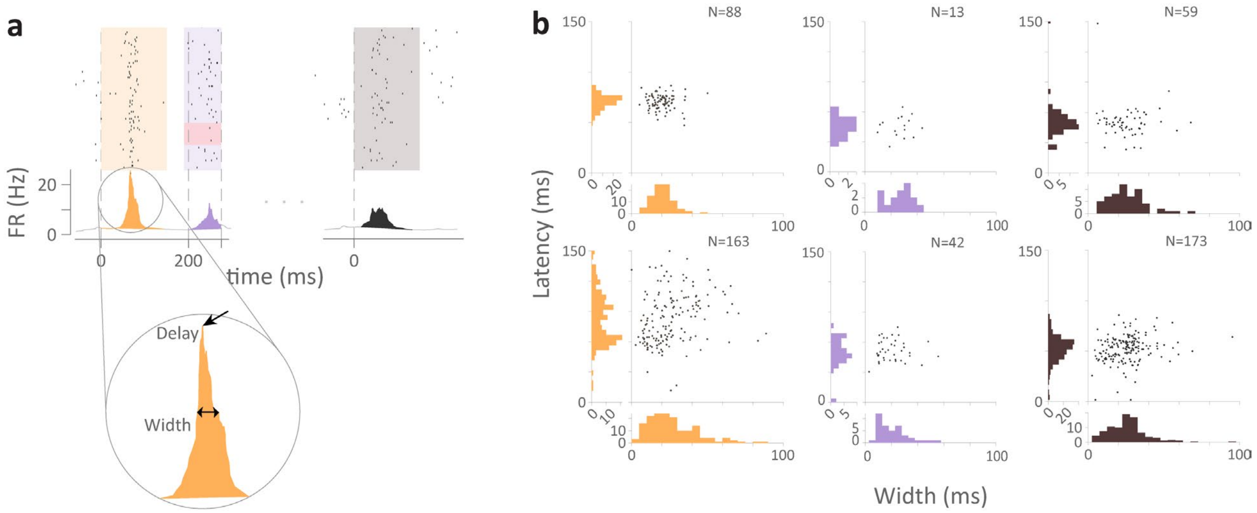

Supplementary Figure 3: Characterization of the width and latency of complex spike signals. a) CSpk latency is computed as the interval (between event onset and spike time) at which distribution peak of all individual spikes occurs. Width is computed as the time elapsed between the $T_{halfmax}$ of the rising and decay phases of the instantaneous firing rate. b) Examples of widths vs. delays computed for all CSpks in a given mouse. $CSpk_{Light}$, (orange) $CSpk_{Prior}$ (purple), and $CSpk_{Airpuff}$ (black).

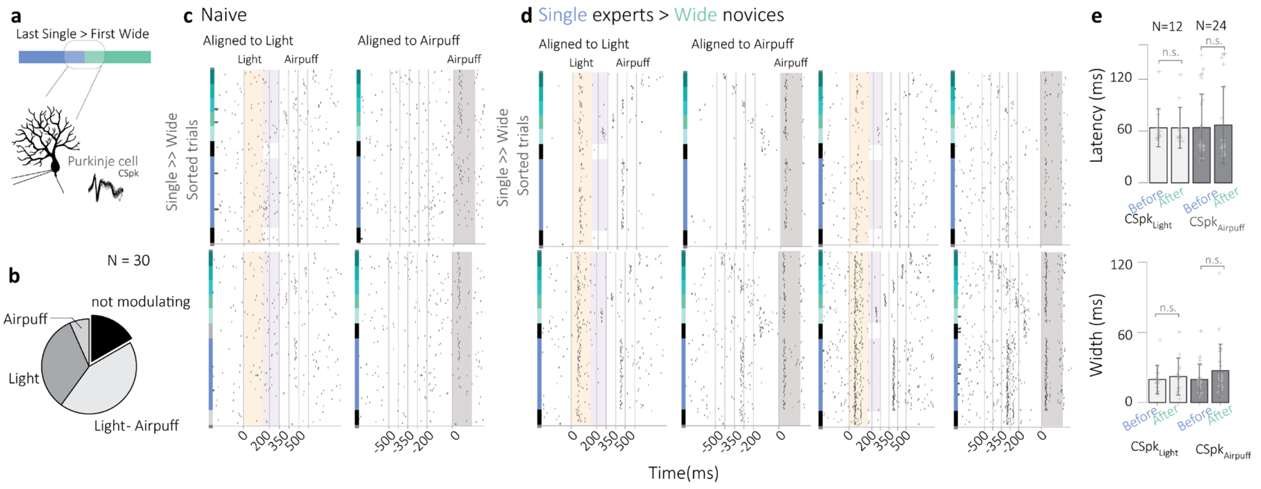

Supplementary Figure 4: Purkinje cell complex spikes in the Wide condition. a) Purkinje cell complex spikes were recorded in Naive subjects and in switch mice as they transitioned from Single to Wide. b) Percentage modulation and the functional categories of complex spikes identified in the mice that switched from Single to Wide. No $CSpk_{prior}$ were found. c) Purkinje cell CSpks recorded in Naive mice as they switched from the last Single session to the first Wide session. Left: Rasters are aligned to the Light, orange and purple indicate the windows where $CSpk_{light}$ and $CSpk_{prior}$ would be expected. Right: Rasters are aligned to the airpuff. d) Same as c) for Single experts that switch to the first Wide condition. Here, we found a consistent $CSpk_{light}$ but no $CSpk_{prior}$ was observed. e) Comparisons with t-statistics of latency and width for the $CSpk_{light}$ (latency: $t(11) = -0.043$, $p = 0.97$; width: $t(11) = -0.30$, $p = 0.77$), and $CSpk_{airpuff}$ (latency: $t(23) = -1.3$, $p = 0.21$; width: $t(23) = -0.62$, $p = 0.54$) before and after the switch. All t-tests are two-sided. Crosses represent individual values, bars represent averages. Error bars represent standard error.

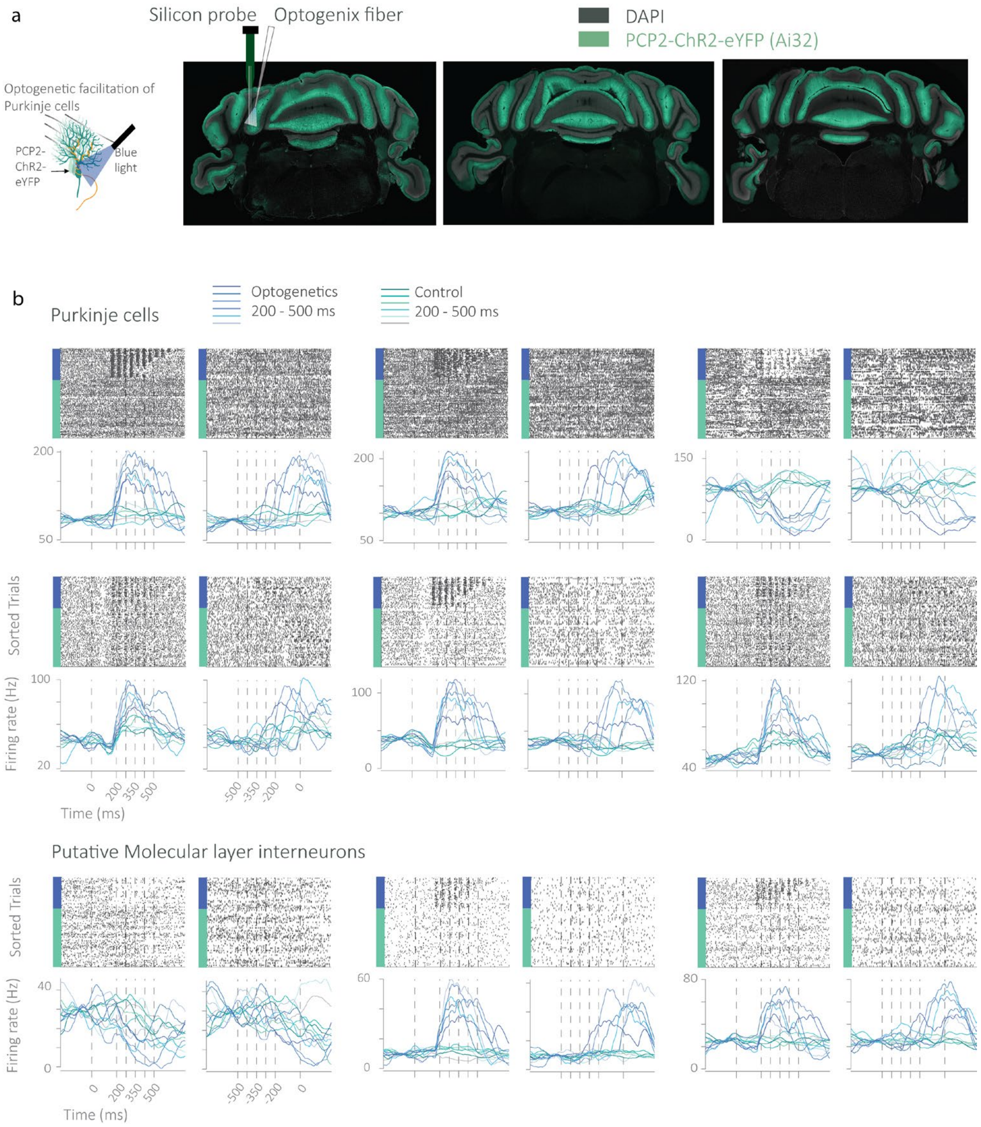

Supplementary Figure 5: Expression of ChR2 and electrophysiology during optogenetics. a) Left: Schematic of optogenetic strategy to acutely stimulate Purkinje cells expressing Channelrhodopsin2 (ChR2) with blue light. Right: Three coronal cross-sections of Ai32 mutants show uniform expression of ChR2 in Purkinje cells (PCP2-Cre-ChR2-eYFP). Electrophysiological recordings were made from lobule simplex where an Optogenix tapered fiber was also implanted. b) Purkinje cell and putative MLI rasters and firing rates over time in the Wide condition during optogenetic trials and control trials within the same session. Optogenetic perturbation was applied at random on 40% of the trials but the rasters have been sorted for visualization.

Supplementary Tables:

Prior Condition	Sample Size (N)	Test Statistic	p-value
a) Between Mice (A_{CR} per mouse based on previous trial type)			
Single	3	$t(2) = 7.68$	$p = 0.01$
Narrow	8	$t(7) = 2.77$	$p = 0.02$
Wide	6	$t(5) = 4.97$	$p < 0.01$
Bimodal	5	$t(4) = 1.88$	$p = 0.13$
Switch	4	$t(3) = 8.30$	$p < 0.01$
b) Within Individual Trials (A_{CR} on test trials contingent on previous trial)			
Single	398 trials	$t(397) = 2.27$	$p = 0.02$
Narrow	1557 trials	$t(1556) = 4.38$	$p < 0.001$
Wide	1087 trials	$t(1086) = 3.12$	$p < 0.01$
Bimodal	1305 trials	$t(1304) = 3.48$	$p < 0.001$
Short	425 trials	$t(424) = 2.56$	$p = 0.01$

Supplementary Table 1: a) Comparison of the average A_{CR} on a test trial when the N-1th trial was a paired trial vs. when it was a test trial. We observe an attenuation of A_{CR} on the Nth trial when the previous trial was a test trial. b) Examples of individual mice where this attenuation of A_{CR} can be observed when comparing a paired trial vs. a test trial on the previous trial.

Prior Condition	Measure	Test Statistic	p-value
a) One-way ANOVA results from N-1th interval comparisons in Wide Expert mice (within individuals)			
Wide Expert	Peak Time	F(4,163) = 1.5	p = 0.21
	AUC	F(4, 288) = 1.24	p = 0.30
	A_{CR}	F(4, 652) = 0.782	p = 0.54
	Time of Peak Velocity	F(4, 652) = 0.576	p = 0.68
b) N-1th interval history effects within mouse groups (across mice)			
Narrow (N = 7 mice)	Peak Time	t(6) = 0.51	p = 0.63
	AUC	t(6) = 0.117	p = 0.91
	A_{CR}	t(6) = 1.53	p = 0.18
Wide Expert (N = 4 mice)	Peak Time	t(3) = -0.142	p = 0.90
	AUC	t(3) = -2.03	p = 0.14
	A_{CR}	t(3) = -2.36	p = 0.10

Supplementary Table 2: a) N-1th analysis on different intervals in uncertain distributions: One-way ANOVA results from individual Wide Expert mice reveal no significant differences in AUC, A_{CR}, or time of peak velocity across conditions. b) Comparisons across all interval conditions within mouse groups (Narrow and Wide Expert) show no significant differences in peak time, AUC, or A_{CR} based on the interval condition of the previous trial. These results suggest that the interval presented in a previous paired trial does not significantly influence response metrics within the Wide and Narrow prior.

%EV Switch				%EV Expert					
Single		Wide		Wide		Narrow		Bimodal	
PC1	PC2	PC1	PC2	PC1	PC2	PC1	PC2	PC1	PC2
63	8	63	11	31	13	48	19	57	10
42	17	42	9	38	14	53	11	31	10
38	15	38	17	54	10	23	12	42	8
28	12	28	7	35	11	52	12		
46	14	46	14	70	7	45	19		
59	13	59	8	44	8	50	8		
53	11	53	11	29	7				
45	13	45	11						
49	18	49	7						

Supplementary Table 3: Percentage of explained variance (%EV) captured by the first two principal components (PC1 & 2) derived from the collective neural activity for each mouse in its corresponding experimental condition.

	Mouse H		Mouse I		Mouse D	
$T_{halfmax}$	$t(488) = -5.58$	$p < 0.0001$	$t(702) = -6.03$	$p < 0.0001$	$t(767) = -16.3$	$p < 0.0001$
Peak Vel.	$t(488) = 3.7$	$p < 0.001$	$t(702) = 6.68$	$p < 0.0001$	$t(767) = 8.92$	$p < 0.0001$
A_{CR}	$t(488) = 6.05$	$p < 0.0001$	$t(702) = 7.34$	$p < 0.0001$	$t(767) = 8.92$	$p < 0.0001$
AUC	$t(488) = 1.88$	$p = 0.06$	$t(702) = 5.17$	$p < 0.0001$	$t(767) = 3.41$	$p < 0.001$

Supplementary Table 4: T-statistic and p-values of a two-sided two-sample t-test for the LFADS behavioral metric comparisons for neural and behavioral data from the Single and Wide conditions.

Condition	t(df)	p-value
$CSpk_{Prior}$ - $CSpk_{Light}$	$t(449) = 1.41$	$p = 0.16$
$CSpk_{Prior}$ - $CSpk_{Airpuff}$	$t(377) = -0.65$	$p = 0.52$

Supplementary Table 5: Statistical comparison (two-sided t-test) of complex spike response width (defined as the time between $T_{halfmax}$ points of the firing rate curve) was performed between $CSpk_{Prior}$ vs. $CSpk_{Light}$ and $CSpk_{Prior}$ vs. $CSpk_{Airpuff}$ conditions.

Measure	t(df)	p-value
$CSpk_{light}$ Width	$t(11) = -0.043$	$p = 0.97$
$CSpk_{light}$ Latency	$t(11) = -0.30$	$p = 0.77$
$CSpk_{airpuff}$ Width	$t(23) = -1.3$	$p = 0.21$
$CSpk_{airpuff}$ Latency	$t(23) = -0.62$	$p = 0.54$

Supplementary Table 6: Statistical Comparison of Complex Spike Properties Before vs After the Switch.

Measure	t(df)	p-value
CR Percentage	$t(4) = -2.3$	$p = 0.04$
A_{CR}	$t(4) = -1.98$	$p = 0.06$
AUC	$t(4) = -2.1$	$p = 0.05$

Supplementary Table 7: Effect of Optogenetic Perturbation on Conditioned Response Metrics. Statistical comparison between control and optogenetic trials shows significant decreases in CR percentage, A_{CR}, and AUC.

LFADS hyperparameters	Single	Wide
spikeBinMs	1	1
c_co_dim	0	0
c_batch_size	15	15
c_factors_dim	40	40
c_gen_dim	64	64
c_ic_enc_dim	64	64
c_learning_rate_stop	1.00E-05	1.00E-05
c_kl_ic_weight	0.1	0.1
c_ic_dim	50	50
c_learning_rate_n_to_compare	3	3
useAlignmentMatrix	TRUE	TRUE

Supplementary Table 8: Hyperparameters used to train LFADS models in Single and Wide conditions.

Cell Type	Single (N = 14 mice)	Narrow (N = 6)	Wide (Experts (N = 6) and Switch (N = 13))	Bimodal (N = 5)
pGoC	8	14	32	10
pMLI	162	160	283	77
pMFB	129	131	135	43
Purkinje SSpk	176	237	258	79
Purkinje CSpk	273	329	326	364

Supplementary Table 9: Neuron cell-type counts per prior condition.
